# Overcoming the On‐Target Toxicity in Antibody‐Mediated Therapies via an Indirect Active Targeting Strategy

**DOI:** 10.1002/advs.202206912

**Published:** 2023-01-22

**Authors:** Zhongjie Tang, Xiaoyou Wang, Mei Tang, Jin Wu, Jiexuan Zhang, Xinlong Liu, Feiyan Gao, Yu Fu, Peng Tang, Chong Li

**Affiliations:** ^1^ Medical Research Institute College of Pharmaceutical Sciences Southwest University Chongqing 400715 P. R. China; ^2^ Department of Breast and Thyroid Surgery Southwest Hospital Chongqing 400038 P. R. China

**Keywords:** antibody therapy, breast cancer, cell membrane biomimetic, indirect active targeting, on‐target toxicities

## Abstract

Antibody‐based therapies could be led astray when target receptors are expressed on nontarget sites, and the on‐target toxicity poses critical challenges to clinical applications. Here, a biomimetic indirect active targeting (INTACT) strategy is proposed based on receptor expression disparities between nontarget sites and the targets. By prebinding the antibodies using cell membrane vesicles with appropriate receptor expressions, the INTACT strategy could filter out the interactions on nontarget sites due to their inferior receptor expression, whereas ensure on‐demand release at the targets by competitive binding. The strategy is verified on CD47 antibody, realizing drastic alleviation of its clinically concerned hematotoxicity on a series of animal models including humanized patient‐derived xenograft platforms, accompanied by preferable therapeutic effects. Furthermore, the INTACT strategy proves extensive applicability for various systems including antibody, antibody–drug conjugate, and targeted delivery systems, providing a potential platform refining the specificity for frontier antibody‐related therapies.

## Graphical abstract

By prebinding the antibodies using cell membrane vesicles with appropriate receptor expressions, the indirect active targeting (INTACT) strategy could filter out the interactions on nontarget sites due to inferior receptor expression while ensuring on‐demand release at the targets by competitive binding. This study provides a potential platform for refining the specificity for frontier antibody‐related therapies.

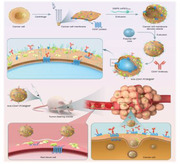

## 1 Introduction

A critical biological foundation for the targeting of many antibodies is their specific cell membrane receptors which, in ideal circumstances, should have high and exclusive expressions on the target,^[^ ^1^ ^]^ whereas are more frequently distributed on numerous nontarget cells/tissues in practice. Such insufficient expression disparity on target and nontarget sites results in an “on‐target” effect (known as the “on‐target off‐tumor” effect in antitumor treatments^[^ ^2^ ^]^) with target‐mediated toxicities, restraining the otherwise promising clinical application of antibody‐related therapies.^[^ ^3^ ^]^ CD47, as a highlighted target for anticancer immune checkpoint blockade therapies,^[^ ^4^ ^]^ has been challenged by on‐target erythrotoxicity, platelet toxicity, and T‐cell toxicity due to unwanted target recognition and blocking. Several clinical trials were terminated owing to nonnegligible hematotoxicities,^[^ ^5^ ^]^ and concerns remain on their potential disturbance of immunological functions as well.^[^ ^6^ ^]^ As another example, cardiopulmonary and skin toxicities have been observed in therapies targeting human epidermal growth factor receptor 2 (HER2) and epidermal growth factor receptor (EGFR).^[^ ^7^ ^]^ Similar concerns about on‐target toxicities also widely exist in antibody‐mediated drug deliveries,^[^ ^8^ ^]^ although off‐target toxicities have already been largely relieved by their active targeting capabilities.

Emerging premasking strategies prove a potential solution with antibody blockage in systemic circulation and specific exposure at target sites,^[^ ^3^, ^9^ ^],^ e.g., probodies. Several clinical trials are ongoing for probodies targeting CTLA‐4, CD71, and PD‐L1, etc.,^[^ ^10^ ^]^ whereas important challenges remain on the simultaneous preservation of efficacy together with the effective protection from toxicities, and relevant developments are still at early stages.^[^ ^11^ ^]^ Meanwhile, receptor expression disparities between target and nontarget sites might provide another inception for mild and efficient noncovalent solutions. Derived from antibody technologies, chimeric antigen receptor (CAR)‐T cell therapies are also faced with on‐target toxicities, and Lim et al. reported an ultrasensitive threshold technique sensing antigen densities through optimization and modification of CAR‐T system, sharply discriminating high‐receptor‐expression targets from low‐receptor‐expression sites thus resolving the on‐target off‐tumor effects.^[^ ^7c^ ^]^ Actually, in the context of binding competitiveness, receptor numbers add to the advantage of high‐receptor‐expression cells in ligand recognition and selective binding compared with low‐receptor‐expression ones, and on‐target toxicity on normal sites might therefore be relieved by preferable binding. Currently, cell membranes and their fragments with various membrane proteins (functional receptors) have been widely utilized in biomedical fields. For example, membranes with high EGFR expressions were adopted as the stationary phase of cellular membrane affinity chromatography for ligand screening,^[^ ^12^ ^]^ and tumor cell membranes with tumor‐homing membrane proteins have helped in the construction of actively targeting delivery systems.^[^ ^13^ ^]^ Hence, cell membranes with appropriate receptor levels see prospects as prebinding modules on antibody or antibody‐mediated drug targeting, which could not be substituted by low‐expression receptors at nontarget sites yet could competitively dissociate on the target sites.

Therefore, we propose an indirect active targeting (INTACT) strategy circumventing the on‐target toxic effect and advancing the efforts in effect‐enhancing and toxicity‐reducing. The system was first verified on CD47 antibody. Accordingly, both cell lines and patient‐derived cells with high CD47 expressions were adopted in the construction of the INTACT system, and CD47 antibody was prebound with these cell membranes. Effective shielding from on‐target toxicities and enhanced therapeutic effects was demonstrated on multiple models including single cell, microfluidic chip, and patient‐derived xenograft (PDX) on humanized mice models, as well as in diverse delivery systems including antibody itself, antibody–drug conjugate (ADC), and antibody‐mediated targeted drug delivery system, and the versatility of the strategy was further verified on antibody targeting HER2. The results proved preferable targeting and therapeutic effect, as well as drastically alleviated toxicity and reduced risks of pathogen infection, a fatal threat to patients receiving first‐line anticancer treatments such as chemotherapy, radiotherapy, and immunotherapy.

## 2 Results

### 2.1 Preparation and Characterization of the INTACT System with Required CD47 Antibody Loading

**Scheme 1:**
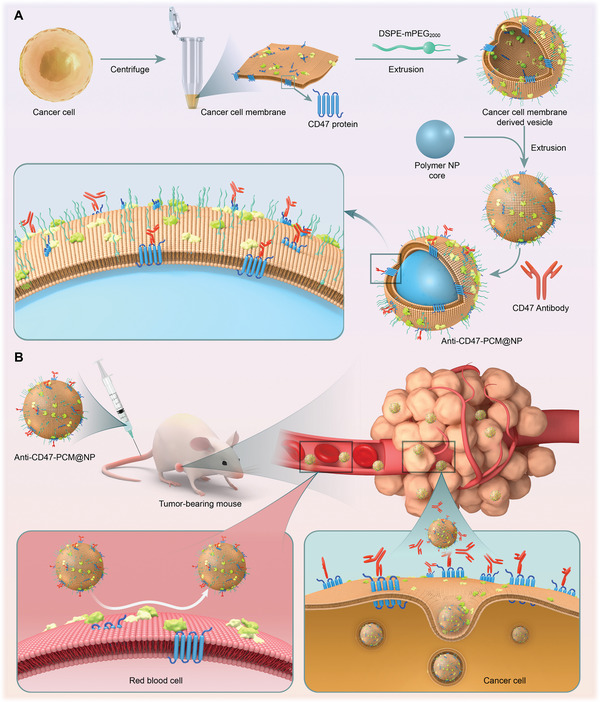
Schematic illustration of the indirect active targeting (INTACT) strategy. A) The coating of the target cell membrane with appropriate receptor expression levels achieves satisfactory antibody loading in the INTACT system. B) The INTACT system distinguishes target cells from low receptor expression nontarget cells, circumventing the on‐target toxicity and advancing the efforts in comprehensive therapeutic performance and toxicity‐reducing.

This CD47 antibody delivery system was constructed by sequential assembly with precise quantification. Mouse breast cancer cell 4T1, a CD47‐positive cell line as reported,^[^ ^14^ ^]^ was selected as the target cell, and its cell membrane (CM) was modified with DSPE‐mPEG_2000_ (forming PCM) and coated on the PLGA nanoparticle core (NP) by extrusion (forming PCM@NP), then the CD47 antibodies were bound to the surface of PCM@NP by incubation (forming anti‐CD47‐PCM@NP) (**Scheme** **1**). The CD47 expressions in a library of cancer cell lines were quantified by Quantum MESF (Molecules of Equivalent Soluble Fluorochrome) microspheres,^[^ ^15^ ^]^ demonstrating the high CD47 expression (344056 ± 21945 CD47 molecules per cell) of the 4T1 cells. The CD47‐expressing on the nontarget red blood cells (RBC) and platelets (PLT) were calculated as 13180 ± 3280 and 33556 ± 5250 CD47 molecules per cell, which were less than 10% of the target 4T1 cells^[^ ^16^ ^]^(Figure S1, Supporting Information).

**Figure 1:**
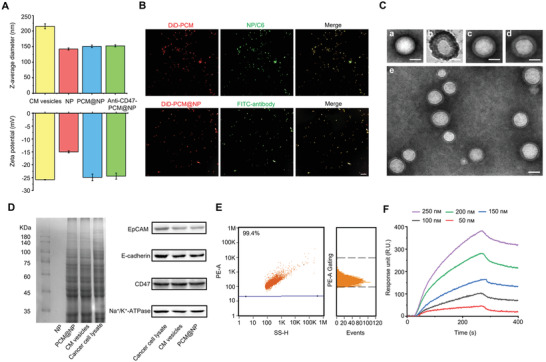
Preparation and characterization of anti‐CD47‐PCM@NP. A) Hydrodynamic size and zeta potential of CM vesicles, PLGA cores (NP), PCM@NP, and anti‐CD47‐PCM@NP. Data are means ± SD (*n* = 3). B) Colocalization of NP/C6 (green) with DiD‐PCM (red), and the colocalization of FITC‐antibody (green) with DiD‐PCM@NP (red), both assessed by confocal laser scanning microscope (CLSM) (scale bar = 5 µm). C) Transmission electron micrographs of (a) NP, (b) CM vesicle, (c) PCM@NP, (d) Anti‐CD47‐PCM@NP, and (e) multiple anti‐CD47‐PCM@NP. All scale bars = 100 nm. D) SDS‐PAGE protein analysis of NP, PCM@NP, CM vesicles, and cancer cell lysate. Samples were tested at equal protein concentrations. CD47 protein and membrane‐specific protein on the cancer cell membrane were efficiently retained on the extracted membrane vesicles and the PCM@NP, detected by western blot. E) Determination of the antibody labeled by PE loaded on the surface of anti‐CD47‐PCM@NP by flow nanoanalyzer. F) The binding affinity of the antibody to the CM vesicles by surface plasmon resonance (SPR).

To realize precise system construction in a quantitative manner, the total phospholipid content of CM was first determined by enzyme‐linked immunosorbent assay (ELISA), and the CM extracted from 1 × 10^9^ 4T1 cells contained a phospholipid content of 4.52 mg (Figure S2, Supporting Information). This amount of CM modified with 3% (molar ratio) DSPE‐mPEG_2000_ was adopted to coat approximately 2.88 mg PLGA NP, and antibodies were bound to the consequent structure by incubation, producing 1.67 × 10^13^ nanoparticles measured by a high‐sensitivity Flow NanoAnalyzer (Figure S3, Supporting Information). The successful construction of the INTACT system (anti‐CD47‐PCM@NP) was verified through a series of physicochemical and biochemical analyses (**Figure** **1**A–D and Figures S4 and S5 and Table S1, Supporting Information), and the antibody proved readily loaded through the antibody‐receptor interactions with membrane CD47. In particular, the antibody loading in 1 mL anti‐CD47‐PCM@NP was 49.7 µg, i.e., an average of 51.6 antibodies was loaded on each anti‐CD47‐PCM@NP (Figure 1E and Figure S6, Supporting Information). The dissociation time of the antibody from CM vesicles obtained by *K*_off_ (1.01 × 10^−4^ s^−1^) conversion was as long as 9901 s (Figure 1G and Figure S7, Supporting Information) validated by surface plasmon resonance (SPR), further confirming the satisfactory loading stability of CD47 antibody.

### 2.2 The INTACT System Successfully Retained the Targeting Function of the Parent Cell Membrane and Distinguished Target Cells from CD47‐Expressing Nontarget Cells

**Figure 2:**
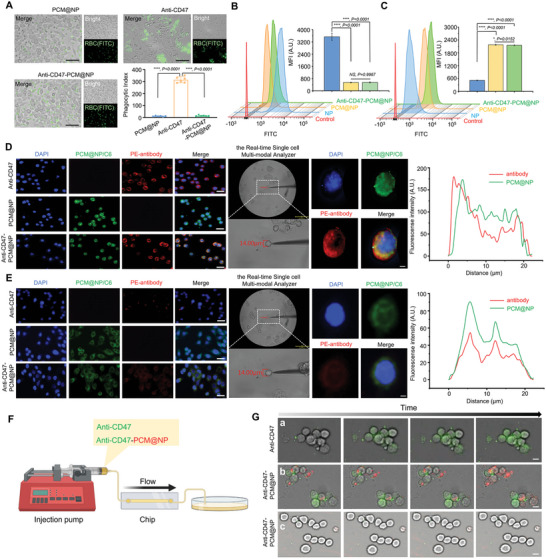
Anti‐CD47‐PCM@NP effectively distinguished target cells from CD47‐expressing nontarget cells in vitro through indirect active targeting. A) Anti‐CD47‐PCM@NP avoided the blocking of CD47 on RBC and subsequent phagocytosis thus circumvented the on‐target toxicity of free anti‐CD47 towards RBC through the INTACT strategy. Scale bar = 50 µm. B,C) PCM@NP and anti‐CD47‐PCM@NP efficiently escaped the capture by B) macrophages with enhanced and parallel cellular uptake by C) target 4T1 cells, measured by flow cytometry. The antibody selectively dissociated from the carrier PCM@NP at the presence of 4T1 cells with high expression of D) CD47 in contrast to E) CD47^−/−^ 4T1 cells, shown by colocalization images and distribution map of PE‐antibody (red) and PCM@NP/C6 (green). Scale bar = 20 µm (multi‐cell images), 2 µm (single‐cell images). F) The schematic diagram of the microfluidic device. The tumor cells were cultured in the cavity of the microfluidic chip till adherence, and then exposed to flowing anti‐CD47 or anti‐CD47‐PCM@NP, and fluorescent images were captured at predetermined time points. G) Free anti‐CD47 sufficiently bound to the surface of 4T1 cells with high expression of CD47 (a). The antibody dissociated from PCM@NP at the presence of 4T1 cells (b), in contrast with CD47^−/−^ 4T1 group (c) (antibody labeled with FITC, green. PCM@NP labeled with DiD, red). Scale bar = 10 µm. Original movies are shown in Movie S1 (Supporting Information) (a), Movie S2 (Supporting Information) (b), and Movie S3 (Supporting Information) (c), respectively. Data are presented as mean ± SD (*n* = 3). (*p < 0.05, **p < 0.01, ***p < 0.001, ****p < 0.0001; NS represents non‐significance).

Two main challenges have been posed to the delivery of CD47 antibody based on its on‐target toxicity: 1) reducing the unwanted release and activity at nontarget sites, and 2) realizing on‐demand release and maintaining antibody activity at target sites. The INTACT system has been verified accordingly. First, the INTACT system achieved substantial bypassing of the nontarget cells. In comparison with the significant phagocytosis of RBC by mouse bone marrow‐derived macrophages (BMDM) in CD47 antibody (anti‐CD47) group due to CD47 blocking, anti‐CD47‐PCM@NP realized comparable RBC biocompatibility with blank PCM@NP, protecting RBC from unwanted antibody release and CD47 blocking (**Figure** **2**A). The INTACT system itself successfully escaped from the immunocapture of the RAW 264.7 macrophages as well (Figure 2B and Figure S8A, Supporting Information). Second, parallel 4T1‐targeting ability with PCM@NP was demonstrated in anti‐CD47‐PCM@NP, which was 3.9‐fold superior to NP, demonstrating the inherited tumor‐homing function from the parent cell membrane (Figure 2C and Figure S8B, Supporting Information), and the precisely on‐demand antibody release was subsequently validated using 4T1 homologous cell line with low CD47 expression (CD47^−/−^ 4T1) as nontarget control (Figure S9, Supporting Information). The above process was accurately visualized by a real‐time single cell multi‐modal analyzer, which enables precise analysis of single living cell with reduced biological noise through nanoprobe electrodes^[^ ^17^ ^]^ (Movies S1–S2 and Figure S10, Supporting Information). Antibody and the carrier PCM@NP in the INTACT system were labeled separately, and antibody (red) accumulated predominantly around the membrane of the target cell, while a large number of PCM@NP (green) entered the cytoplasm, proving effective release of antibodies on encountering target cells due to the competitive binding of the highly‐expressed CD47 (Figure 2D). For nontarget CD47^−/−^ 4T1 cells, no significant antibody release was observed, and only a small amount of anti‐CD47‐PCM@NP was internalized with antibody and PCM@NP maintaining their co‐localization state (Figure 2E).

The precise target recognition of the INTACT formulation was further real‐time validated in a microfluidic system that closely mimics the actual in vivo flowing conditions^[^ ^18^ ^]^ (Figure 2F). It proved feasible for free anti‐CD47 in the flowing state to bind to the target cells adsorbed in the cavity of the microfluidic chip (Figure 2G‐a and Movie S3, Supporting Information), and for anti‐CD47‐PCM@NP, antibodies were effectively released and accumulated around the target cells membrane, accompanied by substantial internalization of the dissociated PCM@NP (Figure 2G‐b and Movie S4, Supporting Information). In contrast, no significant surface binding of antibody or PCM@NP internalization was observed on nontarget cells under this circumstance (Figure 2G‐c) and Movie S5, Supporting Information).

**Figure 3:**
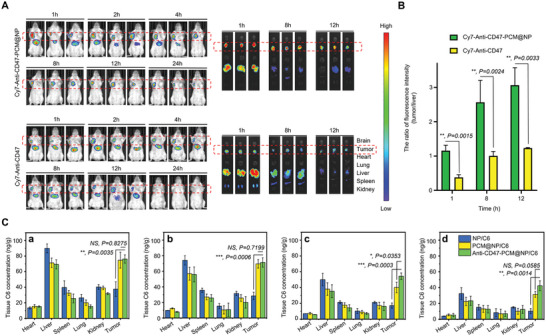
Evaluation of the biological functions of anti‐CD47‐PCM@NP in vivo. A) In vivo and ex vivo targeting ability of anti‐CD47‐PCM@NP and anti‐CD47 in tumor‐bearing mice models determined by live imaging. B) The semiquantitative analysis of the ratio of fluorescence intensity (tumor/liver) of ex vivo imaging. C) In vivo biodistribution of coumarin 6 (C6)‐labeled formulations in tumor‐bearing mice models at (a) 2 h, (b) 4 h, (c) 8 h, and (d) 12 h after i.v. injection of NP/C6, PCM@NP/C6 and anti‐CD47‐PCM@NP/C6. Data are presented as mean ± SD (*n* = 3) (*p < 0.05, **p < 0.01, ***p < 0.001, ****p < 0.0001; NS represents non‐significance).

In vivo and ex vivo imaging further demonstrated the tumor‐targeting ability of anti‐CD47‐PCM@NP, with enhanced tumor accumulation of both the antibody and the nanoparticle. As shown in **Figure** **3**A,B and Figure S11 (Supporting Information), the tumor accumulation of Cy7‐labeled antibody in anti‐CD47‐PCM@NP was over two times greater than the anti‐CD47 group on the observed time points, confirmed by fluorescent imaging of tissue (tumor, heart, lung, liver, spleen, and kidney) slices (Figure S12, Supporting Information) and tissue distribution analysis (Figure 3C). In the pharmacokinetics study, the tumor accumulation of the DiD‐labeled nanoparticle core in the PCM@NP/DiD and anti‐CD47‐PCM@NP/DiD groups was also efficiently prolonged compared to the NP/DiD group (Figure S13, Supporting Information), which was of considerable significance for its further application and druggability.

### 2.3 The INTACT System Exhibited Potent Antitumor Efficacy by Precisely Targeting the CD47 Immune Checkpoint

**Figure 4:**
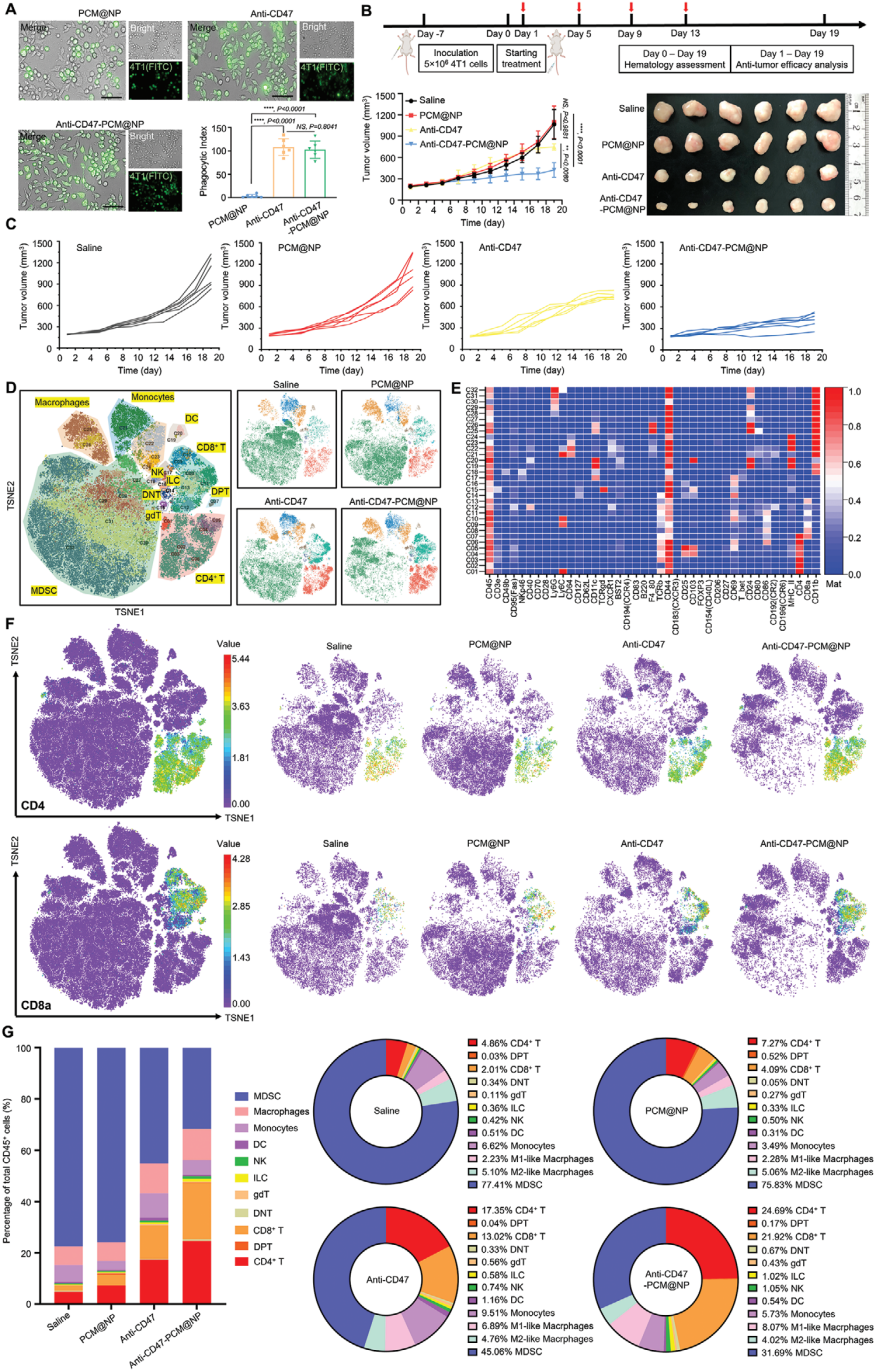
Antitumor efficacy of anti‐CD47‐PCM@NP and mechanistic investigation by CyTOF analysis. A) Representative images and phagocytic index of C57BL/6 bone marrow‐derived macrophages (BMDM) phagocytosing tumor cells following treatment with PCM@NP, anti‐CD47, and anti‐CD47‐PCM@NP. Scale bar = 50 µm. B) Timeline of the anti‐tumor efficacy study on tumor‐bearing mice (red arrows indicate intravenous administrations), and average tumor growth curves and picture of tumor tissues after the treatment. C) Individual tumor growth curves in each group. D) viSNE plot of intratumoral cells in tumor tissues after treatment with saline, PCM@NP, anti‐CD47, anti‐CD47‐PCM@NP and all groups merged. E) Heat map of the surface molecule and functional molecule expression of different subsets of immune cells in tumor tissues from all groups merged. F) tSNE visualization of all samples with the expression of CD4 and CD8a respectively. G) Percentage of cells in each cluster after treatment from each group. Data represented as mean ± SD (*n* = 6). (*p < 0.05, **p < 0.01, ***p < 0.001, ****p < 0.0001; NS represents nonsignificance).

Anti‐CD47‐PCM@NP, with the smart blockade of CD47‐SIRP*α* signaling in the target tumor cells, could induce efficient immunocapture and antitumor efficacy. The phagocytosed number of 4T1 tumor cells by 100 macrophages was quantified in vitro, and equivalent phagocytose (>100 4T1 cells) was induced by anti‐CD47‐PCM@NP compared with direct treatment of anti‐CD47, in contrast to the single‐digit uptake in the PCM@NP group (**Figure** **4**A and Figure S14, Supporting Information). To verify the maximum antitumor potential in vivo, the medication on 4T1 subcutaneous xenograft mice model was not started until the tumor volume in reached ≈200 mm^3^, and mice were dosed (2.5 mg antibody per kg) on days 1, 5, 9, and 13, and sacrificed on day 19. Anti‐CD47‐PCM@NP exhibited substantial suppressions on tumor growth, realizing over 40% reduction of average tumor volume compared with the anti‐CD47 group (Figure 4B,C). Consistent results were observed in the inhibition of malignant proliferation and the immune cell infiltration in tumor tissues by immunohistochemistry (IHC) and immunofluorescence (IF) assays (Figure S15, Supporting Information). Mass cytometry (CyTOF) technology, a plasma‐based single‐cell analysis tool combining mass spectrometry with inductively coupled plasma and flow cytometry, was further utilized to clarify the therapeutic mechanisms. In anti‐CD47‐PCM@NP treatment group, the number of functional immune cells such as CD4^+^ T cells, CD8^+^ T cells, NK cells, and M1‐TAM increased significantly, while the numbers of suppressive immune cells such as M2‐TAM, monocytes, and MDSC have been greatly reduced (Figure 4D–G, Figure S16 and Tables S2 and S3, Supporting Information), suggesting enhanced antitumor immune function of anti‐CD47‐PCM@NP.

The INTACT system further exhibited similar superiorities on a patient‐derived xenograft (PDX) triple‐negative breast cancer tumor model on humanized mice (HuNSG) platform supporting translational researches (Figure S17, Supporting Information). Anti‐CD47‐PCM@NP exhibited significantly greater suppressions on the average tumor sizes compared to the anti‐CD47 group and PCM@NP control group (Figure S18, Supporting Information), with an approximately twofold tumor inhibition rate compared with the anti‐CD47 group on day 19.

### 2.4 The INTACT Strategy Comprehensively Relieved On‐Target Toxicity and Infection Risks, with Improved Prognosis

**Figure 5:**
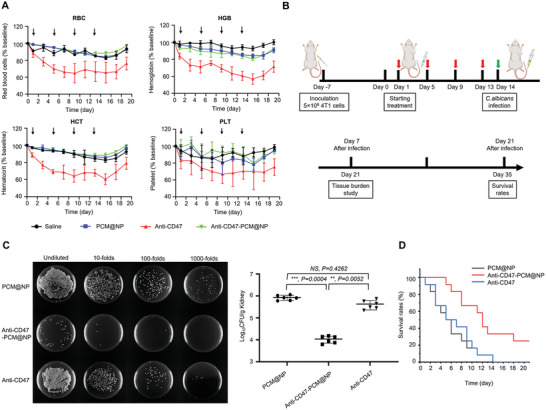
The INTACT strategy efficiently delivers antibodies to tumors with reduced in vivo toxicity. A) Anti‐CD47‐PCM@NP exhibited no significant influence on red blood cell (RBC), hemoglobin (HGB), hematocrit (HCT), and platelet (PLT). Data represented as mean ± SD (*n* = 3). B–D) Anti‐CD47‐PCM@NP relieved the occurrence of fungal infection during antitumor treatment. B) Experimental timeline and treatments in tumor‐bearing mice (arrows indicate intravenous administrations). At day 14, mice were infected with *C. albicans* via tail vein injection. C) Colony‐forming units (CFU) on day 7 in the kidneys of infected mouse models (*n* = 6). D) The survival rates of infected mice with different treatments (*n* = 12). Data represented as mean ± SD. (*p < 0.05, **p < 0.01, ***p < 0.001, ****p < 0.0001; NS represents non‐significance).

Comprehensive biocompatibility assessments were carried out during the in vivo antitumor efficacy, in addition to routine safety inspection experiments such as mouse body weight changes and H&E (hematoxylin and eosin) staining (Figures S19 and S20, Supporting Information). As a highlighted concern in the clinical application of CD47 therapies, hematotoxicity has become the main focus of the safety inspection. Encouragingly, the INTACT system, in addition to its therapeutic effect, exhibited robust in vivo protection from hematological toxicities. The changes in red blood cell (RBC), hemoglobin (HGB), hematocrit (HCT), and platelet (PLT) were monitored every other day during the treatment course, and severe hematotoxicity was validated in anti‐CD47 group with approximately 30–40% reductions of all the above indicators. In contrast, no significant abnormities were observed in anti‐CD47‐PCM@NP group (**Figure** **5**A and Table S4, Supporting Information), demonstrating sufficient relief of the on‐target hematotoxicity by the INTACT strategy.

Pathogen infection proves a nonnegligible cause of mortality in cancer patients with immunodeficiency. As an important marker in immune responses, CD47 plays a controversial role in the infection process, raising potential concerns for interferences during CD47‐targeting therapies. Therefore, the influence of the INTACT strategy on the risk of pathogen infections was explored on a 4T1 tumor‐bearing model mice, and *Candida albicans*, which is among the most common and life‐threatening pathogens in concurrent infections, was adopted as a model pathogen (Figure 5B). Teeming presence of *C. albicans* was observed in the PCM@NP group and the anti‐CD47 group after infection, whereas anti‐CD47‐PCM@NP group manifested two orders of magnitude fewer colony‐forming units (CFU) (Figure 5C and Figure S21, Supporting Information). In addition, the mean survival time of the anti‐CD47‐PCM@NP group after infection (14.7 d) was significantly longer than that of the anti‐CD47 (7.8 d) and PCM@NP (6.9 d) groups (Figure 5D). The above results proved the superior biocompatibility of the INTACT strategy with reduced on‐target toxicity of current CD47‐targeting therapies and relieved concurrent infection burden as well.

### 2.5 The INTACT Strategy Carries Potential as a Platform for Versatile Antibody‐Based Systems

**Figure 6:**
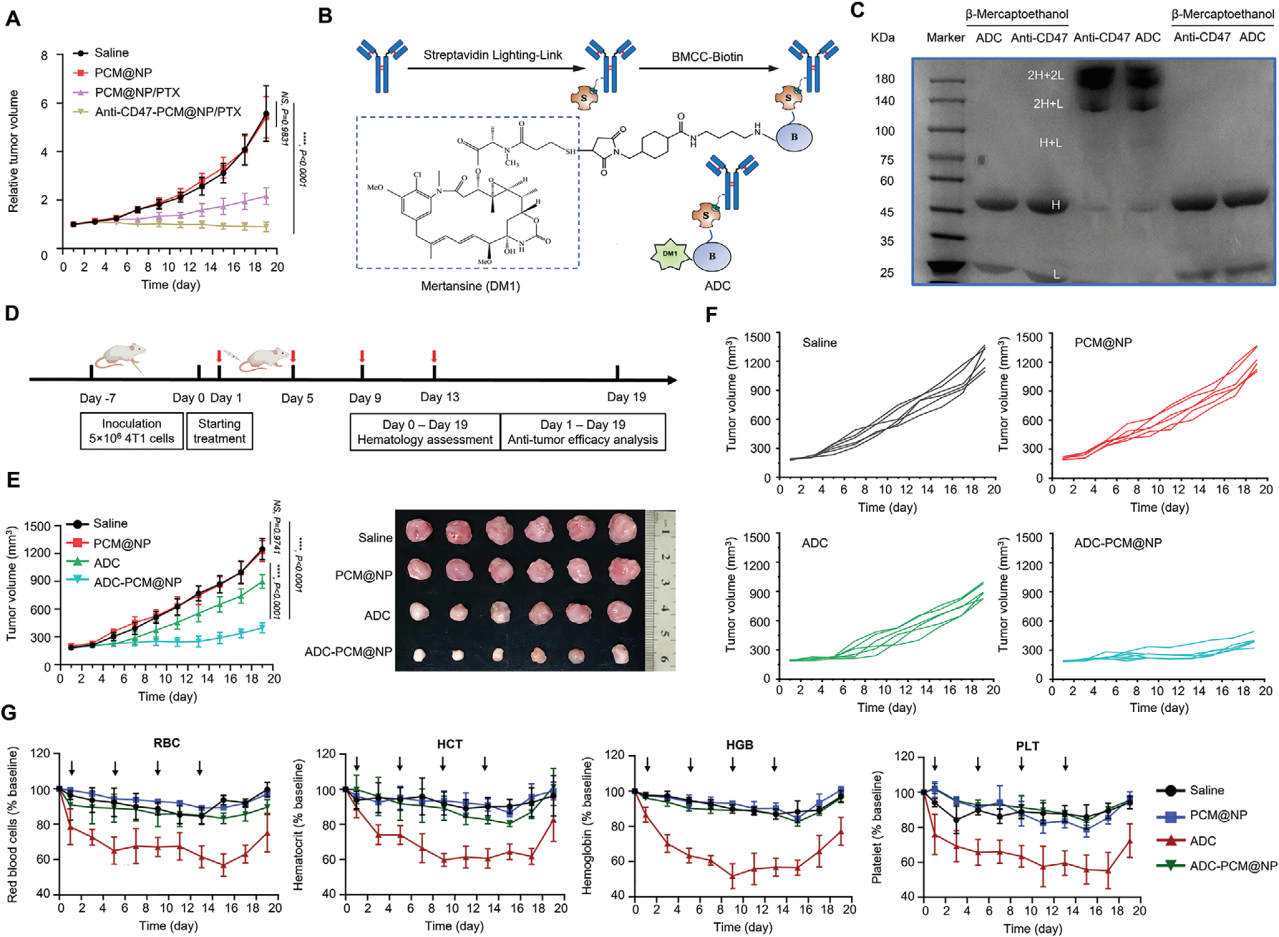
The INTACT strategy is adaptive to multiple antibody‐based systems. A) Relative tumor volume growth with anti‐CD47‐PCM@NP/PTX treatment (*n* = 6). B–G) The INTACT therapy refined the targeting precision of ADC. B) The diagram of ADC construction: Anti‐CD47 was modified with streptavidin and conjugated with DM1 via the crosslinker BMCC‐biotin. C) The conjugation of ADC was confirmed with SDS‐PAGE. D) Experimental timeline for the anti‐tumor efficacy study and hematology assessments of ADC‐PCM@NP (red arrows indicate intravenous administrations). E) Average tumor growth curves, and picture of the tumor tissues after the treatment (*n* = 6). F) Individual tumor growth curves in each group (*n* = 6). G) Hematology assessments of red blood cell (RBC), hemoglobin (HGB), hematocrit (HCT), and platelet (PLT) (*n* = 3). Data represented as mean ± SD. (*p < 0.05, **p < 0.01, ***p < 0.001, ****p < 0.0001; NS represents non‐significance).

Besides the precise delivery of antibodies, the nanoparticle core of the INTACT system also enables the targeted delivery of small‐molecule drugs for combinational use. As an example, paclitaxel (PTX)‐loaded anti‐CD47‐PCM@NP/PTX was prepared with approximately 90% encapsulation efficacy, and satisfactory stability and release behaviors (Figure S22, Supporting Information). In vivo antitumor assay demonstrated the most prominent tumor inhibition effect of anti‐CD47‐PCM@NP/PTX which suppressed the tumor volume from ≈200 mm^3^ to ≈160 mm^3^, proving consistent tendency with in vitro IC_50_ results (**Figure** **6**A, Figure S23 and Table S5, Supporting Information). Increased immune cell infiltration was also observed within the tumor tissue (Figure S24, Supporting Information).

Apart from the delivery of antibodies themselves, the INTACT strategy was further verified to tackle the on‐target toxicity of the highlighted antibody–drug conjugate (ADC) systems.^[^ ^19^ ^]^ A model ADC was constructed by conjugating streptavidin‐labeled anti‐CD47 with a biotinylated, FDA‐approved cytotoxic payload mertansine (DM1) which blocks microtubulin polymerization and suppresses triple‐negative breast cancer^[^ ^20^ ^]^ (Figure 6B,C). The model ADC was loaded onto the PCM@NP of the INTACT platform with similar process, generating ADC‐PCM@NP. In vivo antitumor efficacy was assessed with 2.5 mg antibody per kg concentration (Figure 6D), demonstrating more substantial anti‐tumorantitumor efficacy of ADC‐PCM@NP compared with the ADC group (Figure 6E,F and Figure S25, Supporting Information), as well as satisfactory biocompatibility and potent relief of hematotoxicities (Figure 6G, Figures S26 and S27, and Table S6, Supporting Information).

The functionalization of liposomes with monoclonal antibodies (forming actively targeting liposomes) has emerged as a promising strategy for the targeted delivery towards cells overexpressing relevant receptors,^[^ ^21^ ^]^ and such systems could also benefit from the INTACT strategy. A CD47 antibody‐conjugated liposome was developed, and cancer cell membrane vesicles (CMV) were adopted to reversibly block the CD47 antibody to relieve the on‐target effect, forming CMV‐anti‐CD47‐Lip (Figure S28A, Supporting Information). The system maintained its recognition of target cells (4T1) from nontarget cells (CD47^−/−^ 4T1) verified by CLSM. On encountering target cells, efficient shedding of CMV blockage was observed, and the dissociated anti‐CD47‐Lip (green) was internalized by target cells in large quantities. For nontarget cells with low CD47 expression, no significant release of anti‐CD47 was induced (Figure S28B,C, Supporting Information).

Human epidermal growth factor receptor 2 (HER2)‐targeted therapies, e.g., trastuzumab, have revolutionized the treatment of HER2‐positive breast cancer, yet researches were deterred by cardiopulmonary toxicities due to the expression of HER2 in cardiomyocytes and bronchial epithelial cells, and such toxicity issues are even worsened in patients with concurrent chemotherapy.^[^ ^22^ ^]^ Exhilaratingly, the INTACT strategy also realized successful toxicity reduction of HER2 antibodies, indicating extended applicability for antibodies with different targets. Anti‐HER2‐PCM@NP was constructed in a similar process, and TUBO cells overexpressing HER2/neu protein were adopted to establish subcutaneous xenograft mouse models (Figure S29, Supporting Information). Compared with the significantly abnormal levels of aspartate (AST), cardiac troponin‐I (CTN‐I), and lactate dehydrogenase (LDH) induced by free HER2 antibodies (anti‐HER2), anti‐HER2‐PCM@NP system exhibited paralleling biocompatibility with saline group (Figure S30A, Supporting Information). Disordered and loose arrangement of myocardial muscle, nucleolytic, muscle fiber rupture, and vacuolization was observed in anti‐HER2 treated mice by H&E and Masson staining, accompanied by obvious myocardial capillary congestion and myocardial fibrosis, while no obvious pathological changes were found in saline and anti‐HER2‐PCM@NP groups (Figure S30B, Supporting Information). The intra‐alveolar hemorrhage and interstitial fibrosis in the lung tissues caused by anti‐HER2 were also relieved in anti‐HER2‐PCM@NP group (Figure S30C, Supporting Information). These results proved that the application of the INTACT strategy could be extended to diversified antibodies with effective reduction of the in vivo on‐target toxicity.

## 3 Discussion

Recent decades have witnessed the dramatic evolvement of antibodies which made up an inextricable part of biomedical sciences due to their specific target recognition and binding with subsequent biological functions. As a classical representative of the “magic bullet” concept,^[^ ^23^ ^]^ antibodies could both perform precise therapeutic activities themselves^[^ ^24^ ^]^ and provide targeting moieties for other drugs and delivery systems,^[^ ^25^ ^]^ supporting cutting‐edge technologies such as nanobodies,^[^ ^26^ ^]^ intrabodies,^[^ ^27^ ^]^ bi‐ and tri‐specific antibodies,^[^ ^28^ ^]^ antibody–drug conjugates (ADCs) and antibody–oligonucleotide conjugates,^[^ ^29^ ^]^ as well as diversified immunotherapeutic strategies including checkpoint blockade antibodies and chimeric antigen receptors (CAR) T cell therapies,^[^ ^1^, ^7^, ^30^ ^]^ etc. Despite the intense progress, however, reports of insufficient accuracy and subsequent toxicity concerns prove to blunt their clinical applications, e.g., the on‐target adverse reactions in nontarget tissues reported in antibodies and ADCs, and the fatal cytokine release syndrome caused by CAR‐T cell activation, prompting further improvements on their targeting specificity.^[^ ^1^, ^7^, ^31^ ^]^ Although emerging technologies such as probodies^[^ ^32^ ^]^ could provide reduced toxicity through the enzyme‐responsive masking of parent antibodies, alternative solutions with robustly preserved efficacy are still in need.

Currently, several solutions have already been developed to tackle the on‐target toxicities thus advancing CD47 antibodies to the clinics, e.g., controlling the therapeutic dose, differentiating erythrocyte CD47 from tumor cell CD47 according to their distinct modifications and conformations, etc.,^[^ ^5^, ^33^ ^]^ whereas much efforts are needed when adapting case‐by‐case solutions to other antibodies and antibody‐based systems. The INTACT strategy, however, provides a potential platform which could be further extended to varied antibody types, delivery systems, antibody‐involved combinational therapies, and therapeutics concerning ligand‐receptor interactions for precise regulation, such as peptides, aptamers, CAR‐T systems, as well as various active‐targeting delivery systems since the on‐target toxicity and competitive binding widely exist in their applications.^[^ ^7c^ ^]^ It is also worth noting that different cloaking integrities of cell membrane on nanoparticles are reported to influence their cellular internalization mechanisms, and therefore it might be necessary to further investigate the impact of antibody prebinding densities on the cellular internalization of these biomimetic delivery systems.^[^ ^34^ ^]^ With the development of cell engineering technology, precise regulations of the type and expression levels of membrane receptors could be realized, enabling the individualized design of this strategy. Meanwhile, the innate characteristics and functions of membrane cells further enhance the advantages of such delivery systems, e.g., the targeting ability due to homing effect, and the extra immunologic functions. Furthermore, the prebinding strategy is not merely limited to biomimetic systems, as living cells delivering drugs or drug‐loaded vehicles could also benefit from this strategy.

Resembling the common antitumor therapies such as chemotherapies and radiotherapies,^[^ ^35^ ^]^ the emerging immunotherapies are neither exempted from concerns on increased pathogen infection risks associated with nonnegligible morbidities and mortalities.^[^ ^36^ ^]^ For example, CD47‐targeted therapies might increase the susceptibility to pathogen infections such as *C. albicans*, for CD47 also plays an important role in the regulation of the homeostasis and functions of NK cells.^[^ ^37^ ^]^ Alemtuzumab targeting CD52 was also reported to raise infection rates due to its depletion of CD4 and CD8 T cells, and myelosuppression of the CD20‐targeting rituximab might be responsible for its increased infection risks.^[^ ^38^ ^]^ Accordingly, an infection model was introduced in this study, and a substantially lowered occurrence of infections by the INTACT strategy was verified, which provides a reference for relevant biocompatibility assessments. The treatment of malignancies has always been a comprehensive campaign. Considering the risks of severe infections in cancer patients, it is recommended that more integrated therapeutic assessments were conducted with the occurrence of infection taken into account for antitumor therapies, in the hope of more comprehensive predictions of subsequent medical prognosis.

In summary, our study proposes an indirect active targeting (INTACT) strategy that provides a potential biomimetic solution for on‐target toxic effects. From single‐cell analysis to patient‐derived xenograft models, the INTACT strategy manifested substantial improvement in the specificity and safety, realizing preferable therapeutic effect and drastic toxicity alleviation. The INTACT strategy proved extensively adaptive for multifarious systems based on specific recognitions, including antibody, antibody–drug conjugate, antibody‐mediated drug delivery systems, and antibody‐involved combinational therapies. Frontier targeting therapies and deliveries are commonly questioned with selectivity and subsequent safety issues, and our findings provide a widely applicable strategy for refined specificity and broadened application possibilities.

## 4 Experimental Section

### Materials

PLGA (50:50) (P2191) was supplied from Sigma‐Aldrich Co., Ltd. (Saint Louis, USA). DSPE‐mPEG_2000_ (F01008), and DSPE‐PEG_2000_‐NHS (F02008) were provided by A.V.T. Pharmaceutical Ltd. (Shanghai, China) (purity > 98%). Paclitaxel (HY‐B0015) was provided by MedChemExpress Co., Ltd. (NJ, USA) (purity > 98%). N‐hydroxysuccinimide palmitate (P1162) was supplied from Sigma‐Aldrich Co., Ltd. (Saint Louis, USA). Mertansine (DM1) (HY‐19792) was supplied from MedChemExpress Co., Ltd. (NJ, USA). EZ‐LINK BMCC‐BIOTIN (21900) was provided by Thermo Fisher Scientific Co., Ltd. (MA, USA). Streptavidin Conjugation Kit‐Lightning‐Link (Abcam, ab102921). siRNAs (sense 5’‐3’ CACCGAAGAAAUGUUUGUGAATT; sense 5’‐3’ CCAUACGAAUAAGAGAAUCAUTT) were purchased from Shanghai Sangon Biotechnology Co., Ltd. Quantum R‐PE MESF Medium Level (FCSC827B) was from Bio‐Rad Laboratories, Inc. PE anti‐human/mouse/rat CD47 antibody (E‐AB‐F1016D) was purchased from Elabscience (Wuhan, China). Rabbit anti‐CD47 antibody (bs‐2386R), rabbit anti‐E‐cadherin antibody (bs‐1519R), rabbit anti‐ATPase Na^+^/K^+^ beta 2 antibody (bs‐23413R), rabbit anti‐CD45/AF488 conjugated antibody (bsm‐30095M‐AF488), rabbit anti‐CD8/AF594 conjugated antibody (bs‐0648R‐AF594), and HRP‐labeled goat anti‐rabbit IgG (bs‐40295G‐HRP) were purchased from Bioss Biotechnology Co., Ltd. (Beijing, China) and Absin Bioscience Inc. (Shanghai, China). M‐CSF (P6015) was purchased from Beyotime Biotechnology (Jiangsu, China). InVivomab anti‐mouse CD47 (MIAP301), InVivomab anti‐human/rat HER2 (NEU), and InVivomab anti‐mouse/human CD47 (MIAP410) were supplied from BioXCell (NH, USA). 1,1′‐Dioctadecyl‐3,3,3′,3′‐tetramethylindotricarbocyanine iodide (DiR) (D12731) was obtained from Invitrogen (Carlsbad, CA, USA). 4′,6‐Diamidino‐2‐phenylindole (DAPI) (P36935) and 1,1′‐dioctadecyl‐3,3,3′,3′‐tetramethylindodicarbocyanine,4‐chlorobenzenesulfonate salt (DiD) (D7757) were purchased from Thermo Fisher Scientific (Runcorn, UK). Coumarin‐6 (C6) (C100929) was purchased from Aladdin (Shanghai, China). All the other reagents were analytical grade.

### Strains

*Candida albicans* strain ATCC90028 was acquired from the American Type Culture Collection (ATCC) which was cultured in nutrient‐rich yeast extract‐peptone‐dextrose (YPD) medium at 30 °C.

### Cell Lines

Mouse breast cancer 4T1 cell line was purchased from Procell Life Science & Technology and cultured in DMEM (Gibco) medium. MCF‐7 cells and A549 cells (Procell Life Science & Technology) were cultured in MEM (Gibco) medium containing bovine insulin and Ham's F‐12K medium, respectively. CD47^−/−^ 4T1 cells were transfected with siCD47 using Lipofectamine 2000 (Invitrogen) according to the recommended protocol. Two pairs of siRNA targeting CD47 were designed and their knockdown efficiency was determined by flow cytometry and Western blotting. A20, RM‐1, NCI‐N87, TE‐1, and P388 cells (Procell Life Science & Technology) were cultured in RPMI 1640 medium, respectively. MDA‐MB‐231, MDA‐MB‐468, OVCAR‐3, HepG2, HCT‐116 cells, purchased from KeyGen Biotech, were cultured in DMEM medium. RAW 264.7 cells obtained from the Cell Bank at the Chinese Academy of Sciences, and TUBO cells purchased from Hunan Fenghui Biotechnology were both cultured in DMEM medium. HPAC cells (Procell Life Science & Technology) were cultured in DMEM/F12 medium. All the above cells were cultured in complete medium containing fetal bovine serum (FBS) (Hyclone). Cells were kept in an incubation chamber at 37 °C and 5% CO_2_ with a humidified atmosphere.

### Mice

The female Balb/c mice (5 weeks, 18–22 g), the male C57BL/6 mice (5 weeks, 18–22 g), and Sprague Dawley rats (180–220 g) were obtained from Chongqing Academy of Chinese Materia Medica (Chongqing, China), the female Balb/c nude mice (5 weeks, 18–22 g) and HuNSG mice (5 weeks, 18–22 g) were obtained from Beijing Vital River Laboratory Animal Technology Co., Ltd. (Beijing, China), and were raised in pathogen‐free laboratory animal environment, housed on a 12 h light/dark cycle at 22–24 °C and 30–50% relative humidity. The laboratory animal facility has been accredited by the IACUC (Institutional Animal Care and Use Committee) of Southwest University Laboratory Animal Center (IACUC Issue No. IACUC‐20200120‐02). All animal experiments were conducted under the guidelines of the Ethical Review Committee of experimental animals at the Southwest University of China.

### Cancer Cell Membrane (CM) Derivation

To harvest the membrane, the cells were grown in T‐75 culture flasks to 80–90% confluency, digested with 0.25% Trypsin‐EDTA (1 ×) (Gibco), resuspended in phosphate‐buffered saline (PBS), and washed with PBS for three times by centrifugation at 500 × *g*. Then the cells were suspended in a hypotonic lysis buffer consisting of 20 × 10^‐3^ m tris‐HCl pH = 7.5, 2 × 10^‐3^ m MgCl_2_, 10 × 10^‐3^ m KCl, and protease inhibitor tablet without EDTA per 10 mL solution, and the whole solution was circulated 100 W 20 times using an ultrasonic cell crusher (Scientz‐950E, Scientz Biotechnology Co., Ltd., Ningbo, China), and then centrifuged at 3200 × *g* for 5 min, and the supernatant was collected. The pellets were resuspended in hypotonic lysis buffer, sonicated, and centrifuged again, and the resultant supernatant was also collected. The supernatant was then centrifuged at 10 000 × *g*, and the final sediment was collected and used as purified CM for subsequent experiments. The phospholipid concentration of the CM was determined via a Phospholipid Assay Kit (MAK122, Sigma‐Aldrich) as per the manufacturer's suggested protocol.

### Preparation of Nanoparticles

The PLGA cores (NP) were prepared by oil‐in‐water single emulsion/solvent evaporation technique as reported previously.^[^ ^39^ ^]^ Briefly, 10 mg PLGA (50:50) and 1 mg poloxamer were dissolved in dichloromethane. The aqueous phase 0.25% PVA was passed through 0.45 µm syringe filter to remove undissolved remnants of PVA. The organic phase was added dropwise into 20% (volume ratio) PVA solution, and vortex mixed before a sonication of 400 W 100 s. To evaporate dichloromethane, the solution was stirred for 3 h at 37 °C. Further to load PTX in the PLGA core, an appropriate amount of PTX was added to the organic phase and the nanoparticles were prepared as described above.

The membrane material derived as described above was physically extruded through a 200 nm polycarbonate membrane for 20 times to prepare CM vesicles. PEGylation was realized through postembedding of 3% (molar ratio) DSPE‐mPEG_2000_ on the surface of CM vesicles. The resulting vesicles (PCM) were then coated onto the PLGA core (PCM@NP) by co‐extruding the PEGylated CM vesicles and PLGA core through a 200 nm polycarbonate membrane.

To synthesize anti‐CD47‐PCM@NP, an appropriate amount of antibody was added to the PCM@NP and then incubated at 37 °C for 4 h. The samples were further passed through a Sephadex G‐25 column to remove unbound antibodies. Notably, to further stabilize the anchoring of the antibody, the CD47 antibody (anti‐CD47) was modified with N‐hydroxysuccinimide palmitate. Briefly, anti‐CD47 was dissolved in carbonate buffer solution (pH 9.6), and N‐hydroxysuccinimide palmitate was dissolved in dimethyl sulfoxide (DMSO) at a 1:5 molar ratio. The N‐hydroxysuccinimide palmitate was added dropwise to the anti‐CD47 under stirring, and the mixture was stirred at room temperature for 12 h, then dialyzed against (MW CO 8000–14 000 Da) PBS for 12 h, and the modification ratio of the N‐hydroxysuccinimide palmitate was quantified by the standard ninhydrin method.^[^ ^40^ ^]^

### Characterizations of Nanoparticles

A Zeta Sizer Nano Series (Nano ZS 90, Malvern, UK) was used for dynamic light scattering (DLS) measurement to characterize particle size and zeta potential. The sample was suspended in PBS at 1 mg mL^−1^ concentration, and placed at 37 °C for 72 h. The changes in particle size were measured at different time points (6, 12, 24, 48, 72 h). All measurements were performed in triplicate at room temperature. After negative staining with 1% phosphotungstic acid, the surface morphology of NPs was further studied under a transmission electron microscope (TEM) (Ht7800, HITACHI, Japan).

The fluorescence labeling method was used to verify the co‐localization of PCM and PLGA core. The freshly prepared PCM was labeled with DiD, and the PLGA core was labeled with C6. PCM@NP was prepared according to the above preparation method, and the fluorescence distribution was photographed with a confocal laser scanning microscope (CLSM) (A1R, Nikon, Japan). Furthermore, the fluorescence labeling method was also used to verify the co‐localization of the PCM@NP and the antibody. The PCM@NP was labeled with DiD, and the antibody was labeled with FITC by the Alexa Fluor 488 antibody labeling kit (A20181, ThermoFisher). Anti‐CD47‐PCM@NP was prepared according to the above preparation method, and CLSM was used to obtain the fluorescence distribution.

The quantified number of anti‐CD47‐PCM@NP was assessed with a high‐sensitivity Flow NanoAnalyzer (U30E, NanoFCM, China) providing light scattering and fluorescence analysis of nanoscale particles (7‐1000 nm) in flow cytometer assay, and antibody loading efficiency was also measured.

### SPR Analysis

The OpenSPR system (Nicoya Lifesciences, Waterloo, Canada) was used to verify the binding affinity between the antibodies (N‐hydroxysuccinimide palmitate modified CD47 antibody) and CM vesicles, the anti‐CD47 and CM vesicles, and IgG and Lip (without CD47 protein) were set as the control group. SPR was performed at a constant flow rate of 20 µL min^−1^ at 25 °C. CM vesicles were prepared according to the above method, and then fixed on the Lip‐1 sensor chip (Nicoya Lifesciences, Canada). Then, different concentrations of antibodies and IgG were tested with PBS buffer at a flow rate of 20 µL min^−1^ in SPR. All concentrations were performed in triplicate, and the chip was regenerated by injecting 200 µL of 20 × 10^‐3^ m CHAPS after each test.

### SDS‐PAGE and Western blot (WB) Analysis

For protein characterization by SDS PAGE, all samples were prepared in loading buffer at a final protein concentration of 1 mg mL^−1^, as measured by BCA assay (Pierce BCA Protein Assay Kit, Thermo Scientific). The samples were heated at 95 °C for 10 min to fully denature the protein. Then membrane protein was separated using 10% SDS‐PAGE, stained and eluted with Coomassie brilliant blue, and imaged with a ChemiDoc XRS+ System (Bio‐Rad). For WB analysis, the proteins on SDS‐PAGE were transferred to PVDF membrane with transfer buffer (Invitrogen, CA, USA), and stained with antibodies against EpCAM (abs‐120056, absin), antibodies against E‐cadherin (bs‐1519R, Bioss), antibodies against ATPase Na^+^/K^+^ beta 2 (bs‐23413R, Bioss), CD47 antibody (bs‐2386R, Bioss), and HRP‐labeled goat anti‐rabbit IgG (bs‐40295G‐HRP, Bioss) as the secondary antibodies.

### Analysis of Cell Surface Receptor Expression Levels

Quantum MESF Kits (FCSC827B, Bangs Laboratories, Inc.), which are composed of one blank microsphere and four microspheres labeled with increasing amounts of a specified fluorochrome, were used for the standardization of fluorescence intensity. These microspheres were incubated with the same secondary antibody used to stain the cells, rinsed twice with PBS, and subjected to flow cytometric using FACS (BD biosciences, USA). Cells were harvested using a 0.25% trypsin‐EDTA (1×) (Gibco) 2 d after subculturing at a 1:4 ratio. The cells were diluted at 1× 10^6^ cells per mL in a culture medium and incubated with 1.0 mg mL^−1^ of a PE anti‐human/mouse/rat CD47 antibody (E‐AB‐F1016D, Elabscience) or anti‐Neu/ErbB2/HER2 Antibody (SC‐7301, Santa Cruz Biotechnology) and CoraLite488‐conjugated Affinipure coat anti‐mouse IgG(H+L) (SA00013‐1, Proteintech) for 30 min at 4 °C. The cells were collected by centrifugation at 800 × *g* for 4 min and rinsed with PBS twice and subjected to flow cytometric. The obtained data were analyzed using FlowJo software (Tree Star, Ashland, OR, USA).

### In Vitro Phagocytosis Assay

For the in vitro phagocytosis assays, bone marrow‐derived macrophages (BMDM) were first isolated according to a published protocol with slight modifications.^[^ ^41^ ^]^ In short, after sacrificing C57BL/6 mice and disinfecting the skin with 75% alcohol, the hind legs were cut and placed in a sterile petri dish (35/10 mm) containing sterile, ice‐cold PBS. A 5 mL syringe and a 25 gauge needle were used to flush the bone marrow with PBS. The supernatant bone marrow cells were collected, washed with PBS, and resuspended in BMDM complete medium (100 mL complete medium consisted of 74 mL DMEM + 15 mL M‐CSF (25 ng mL^−1^) + 10 mL FBS + 1 mL penicillin–streptomycin solution) (PS) (100 ×)). The cells were then incubated with 5% CO_2_ at 37 °C, supplemented with fresh medium on the 3rd and 5th days, and harvested on the 7th day. BMDMs were added to a 24‐well tissue culture plate at 5 × 10^4^ per well. RBC/4T1 cells were labeled with 2.5 × 10^‐6^ m carboxyfluorescein succinimidyl ester (CFSE) according to the manufacturer's protocol (Invitrogen). 2 × 10^5^ CFSE‐labeled live RBC/4T1 cells were incubated with PCM@NP, anti‐CD47 (20 µg mL^−1^), N‐hydroxysuccinimide palmitate modified CD47 antibody (20 µg mL^−1^) or anti‐CD47‐PCM@NP (20 µg antibody mL^−1^) for 2–4 h, then add to BMDMs and further incubated for 2 h in serum‐free medium. BMDMs were washed and subsequently imaged with the high content analysis system (Operetta CLS, PerkinElmer, USA). Phagocytosis index was calculated as the number of CFSE+ cells phagocytosed per 100 macrophages.

### In Vitro Cellular Uptake Assay

For fluorescence image observation, RAW264.7 or 4T1 cells were cultured overnight, and C6‐labeled nanoparticles were added to the wells and incubated for another 2 h. After that, the cells were washed thrice with PBS, fixed with 4% paraformaldehyde, washed thrice with PBS again, and the nuclei were stained with DAPI. Images were taken with an inverted fluorescence microscope (DMi8, Leica, Germany) and high content analysis system (Operetta CLS, PerkinElmer, USA). Further to carry out fluorescence colocalization assay in a single living cell, 4T1 and CD47^−/−^ 4T1 cells were incubated separately in a culture dish for 2 h at 37 °C in 5% CO_2_. Anti‐CD47‐PCM@NP was prepared with PE‐labeled antibody and C6‐labeled PCM@NP, and was incubated separately with 4T1 and CD47^−/−^ 4T1 cells. After incubation, fluorescent light guided by the optical micro/nanofiber was used to activate the probe in a single living cell, and the fluorescence intensity was recorded in real time by the real‐time single cell multi‐modal analyzer (Jiangsu Rayme Biotechnology Co., Ltd., China) (Figure S10 and Movies S1 and S2, Supporting Information). The confocal image of the photoactivated single cell was taken by the CLSM with a 60× oil‐immersion objective. For cellular uptake of nanoparticles flow cytometry analysis, RAW264.7 or 4T1 cells were seeded into a 24‐well plate (Corning, USA) and cultured overnight. The C6‐labeled nanoparticles were then added to the wells and incubated for another 2 h. After incubation, cells were washed with PBS for three times, digested with trypsin, and then washed and resuspended with PBS, and the fluorescence was measured using FACS. For each replicate, the average cell fluorescence of 10 000 cells was determined.

### MTT Analysis

4T1 was seeded in a 96‐well plate (5 × 10^3^ cells per well) and cultured overnight. Then the cells were treated with Abraxane (Abr) a marketed PTX formulation, was taken as a control formulation, NP/PTX, PCM@NP/PTX and anti‐CD47‐PCM@NP/PTX for 24 h. Then the medium was replaced with fresh complete medium containing 0.5 mg mL^−1^ MTT. After incubating for 4 h, the medium was removed and the purple formazan crystals were dissolved in 150 µL DMSO. The absorbance of each well at 490 nm was measured using a microplate reader (680, Bio‐Rad, USA).

### Pharmacokinetic Analysis

The female SD rats were randomly divided into 3 groups, and 6 rats in each group were set in parallel. Rats were administered with (I) NP/DiD; (II) PCM@NP/DiD; (III) anti‐CD47‐PCM@NP/DiD through the tail vein, separately, with a dose of 20 µg DiD per kg. At 5 min, 15 min, 30 min, 1 h, 2 h, 4 h, 6 h, 8 h, 12 h, 24 h, 48 h and 72 h, approximately 0.2 mL of blood was collected from the rats’ orbital venous plexus and pretreated with heparin sodium. Samples were centrifuged at 4000 rpm for 10 min, the plasma was collected, methanol was added to precipitate the protein, and the samples were then centrifuged at 10 000 rpm (4 °C) and supernatants were collected. The above process was repeated for three times to fully extract DiD. The collected supernatant was vortexed and stored at ‐20 °C. The samples were rewarmed at 25 °C for 1 h, and the fluorescence intensities were detected (Ex = 595 nm/Em = 670 nm).

### In Vivo and Ex Vivo Nanoparticles Distribution by Live Imaging

DiR‐labeled NP, DiR‐labeled PCM@NP, Cy7‐labeled anti‐CD47‐PCM@NP, and Cy7‐labeled anti‐CD47 were used for real‐time in vivo and ex vivo fluorescence imaging. Balb/c mice were inoculated subcutaneously with a sufficient amount (5 × 10^6^) of 4T1. When the tumor volume reached ≈100 mm^3^, mice were injected with the corresponding preparations through the tail vein. Fluorescence images were recorded by IVIS Lumina LT in vivo imaging system (PerkinElmer, USA) at predetermined time points. For ex vivo imaging, the mice were sacrificed at predetermined time points after administration, and the major organs (heart, liver, spleen, lung, kidney, and brain) and tumor were collected, washed, and imaged by a VISQUE in vivo Smart‐LF System (Vieworks, Korea).

### In Vivo Nanoparticles Distribution

The 4T1 tumor‐bearing mice were injected with C6‐labeled nanoparticles through the tail vein at a dose of 20 µg C6 per kg. At the time points of 2 h, 4 h, 8 h, and 12 h, the mice were sacrificed with carbon dioxide after blood sampling, and the heart, lung, liver, spleen, kidney, and tumor were carefully removed. Then, the blood samples and the weighed tissues were mixed/homogenized with methanol (mass ratio, 1:3) and centrifuged at 10 000 rpm (4 °C) for 30 min. C6 in the sediment was extracted twice with methanol. The supernatant was collected and analyzed with an Infinite F200 pro multi‐mode reader (Tecan, Switzerland) (excitation/emission: 595/665 nm). The C6 concentration in the sample was calculated using a standard curve covering the concentration range of 0.15–20 ng mL^−1^.

Antibody was labeled with FITC, PCM@NP was labeled with DiD, and FITC‐anti‐CD47‐PCM@NP/DiD was prepared accordingly. 4T1 tumor‐bearing mice were randomly divided into 3 groups, FITC‐antibody, PCM@NP/DiD, FITC‐anti‐CD47‐PCM@NP/DiD (100 µL) were injected through the tail vein. Four hours later the mice were sacrificed, and the tumor tissues were obtained by dissection and were embedded in an optimal cutting temperature (OCT) compound. Frozen tissue was sectioned using a Leica CM1850 cryostat (Leica, Germany) at a thickness of 10 µm. The sections were placed on the adhesive slide and kept at room temperature for 30 min, fixed with 4% paraformaldehyde for 15 min, immersed in PBS for three times, stained with DAPI for 5 min, and washed with PBS thrice. The sections were imaged using the High Content Analysis System.

### In Vivo Antitumor Efficacy

For the antitumor efficacy of anti‐CD47‐PCM@NP, 4T1 tumor‐bearing mice were randomly divided into 4 groups (*n* = 6 per group), and different preparations were administered via the tail vein on days 1, 5, 9, and 13, including saline, PCM@NP, anti‐CD47 (2.5 mg per kg), and anti‐CD47‐PCM@NP (2.5 mg antibody per kg). A dosage of 15 mg PTX per kg and 2.5 mg antibody per kg was adopted for the antitumor efficacy of anti‐CD47‐PCM@NP/PTX at the same time. The survival rate was observed for 20 d. The tumor volume [length×(width)^2^/×1/2] and body weight of different groups were measured every 2 d. After the mice of each group were sacrificed, the tumors and harvested organs were subjected to H&E, Masson, IHC, and IF staining. The antitumor efficacy of anti‐CD47‐PCM@NP was further investigated on the patient‐derived xenograft (PDX) models. Briefly, patient breast cancer specimen was harvested within 1 h after surgery according to approved guidelines provided by the Ethics Committee of Southwest Hospital, the Third Military Medical University. In all cases, the patient's informed written consent was obtained at the time of registration. Patient's breast cancer specimens were provided by Department of Breast and Thyroid Surgery, Southwest Hospital. PDX models were generated by the implantation of PDX into HuNSG mice. The study was approved by the Ethics Committee of Southwest Hospital, the Third Military Medical University. (approval number KY2021061). Anti‐CD47‐PCM@NP was prepared by deriving the PDX model cancer cell membranes according to the method described above.

The antitumor efficacies of ADC and HER2 antibody delivered with the INTACT strategy were verified on 4T1‐bearing mouse model in accordance with the above experimental protocol.

### Time‐of‐Flight Mass Spectrometry (CyTOF) Analysis

For CyTOF detection, the fresh tumor tissues of mice in each treatment group were collected and stored in MACS tissue storage solution (Miltenyi Biotec, 130‐100‐008) pre‐chilled to 4 °C. The tumor tissue was then minced and further disintegrated using a tumor isolation kit (Miltenyi Biotech, 130‐096‐730), filtered through a 70 µm cell strainer to obtain a single‐cell suspension, then cell number from each sample was counted, and the viability was measured. For mass cytometry analysis, purified antibodies were obtained from BioLegend, Thermo Fisher, BioRAD or R&D systems and metal‐labeled using the MaxPAR Antibody Labeling Kit (Fluidigm). Details are given in Table S3 (Supporting Information). And titrate the conjugated antibody before use to determine the optimal concentration. For mass cytometry staining, cells from 6 mice per group were pooled in one sample to obtain enough cells for reliable analysis. Cells were washed and stained with 100 µL of cisplatin (250 × 10^‐6^ m, Fluidigm) for 5 min on ice and with surface antibody cocktail after 30 min incubation in Fc receptor blocking solution. After that, cells were washed with FACS buffer (0.5% BSA, 1× in PBS) and fixed overnight in embedding buffer (Maxpar's fixative containing 250 × 10^‐6^ m 191/193Ir, Fluidigm). After washing with FACS buffer and Perm buffer (eBioscience), the intracellular antibody mixture was stained for 30 min. Finally, cells were resuspended in deionized water containing 20% ​​EQ beads (Fluidigm) and recorded on a mass flow cytometer (Helios, Fluidigm). As for CyTOF data analysis, the main steps were as follows: 1) adopting a doublet‐filtering scheme with unique mass‐tagged barcodes^[^ ^42^ ^]^ to decode the data of each sample from raw data; 2) using FlowJo software to exclude debris, dead cells, and doublets, leaving live, single immune cells; 3) applying X‐shift clustering algorithm^[^ ^43^ ^]^ to partition the cells into distinct phenotypes based on marker expression levels; 4) annotating cell type of each cluster according to its marker expression pattern; 5) using dimensionality reduction algorithm tSNE to visualize the high‐dimensional data in two dimensions, and show the distribution of each cluster, marker expression in each group among different sample type; 6) performing statistical analysis on the frequency of annotated cell populations.

### In Vivo Safety Study

Before treatment, peripheral blood was collected from the orbit of the mouse for routine hematology assessment. After the first administration according to the above‐mentioned in vivo antitumor regimen, peripheral blood was collected from the mouse orbit every 2 d for hematology assessment, and the changes in red blood cells (RBC), hemoglobin (HGB), hematocrit (HCT), and platelet (PLT) were analyzed to evaluate the side effects of different dosing regimens. After the in vivo antitumor evaluation of anti‐HER2‐PCM@NP, blood samples of mice in each treatment group were taken after removing the eyeballs, centrifuged at 4000 rpm for 10 min, and the upper serum was collected, and the serum levels of aspartate (AST), cardiac troponin‐I (CTN‐I), and lactate dehydrogenase (LDH) were analyzed with a biochemical index test kit and automatic biochemical analyzer (AU400, Olympus Optical Co., Ltd., Japan).

After the in vivo anti‐CD47‐PCM@NP antitumor efficacy last dose, *Candida albicans* (5×10^5^) was injected through the tail vein. On day 7 after infection, mice were sacrificed, and then the kidney tissues from each group were carefully collected, weighed, and homogenized with sterile saline. The homogenate was continuously diluted by sterile saline, and then 30 mL of the suspension was inoculated onto YPD medium with 1% penicillin–streptomycin (volume ratio, 1:1) and incubated at 30 °C for 48 h to determine CFU per g of kidney tissue. The kidney tissues were collected and fixed in 4% paraformaldehyde solution and embedded with paraffin for sectioning. The samples were stained with periodic acid Schiff (PAS) staining for histopathological examination. The survival rates for all mice were monitored for 21 d after infection.

### Antibody–Drug Conjugate (ADC) Construction and Analysis

The CD47 antibody was conjugated to streptavidin for 3 h using the Lightning‐Link Streptavidin Conjugation Kit (Abcam, ab102921) according to the manufacturer's protocol. Mertansine (DM1) was biotinylated at a molar ratio of 8:1 using EZ‐Link BMCC‐biotin according to the manufacturer's instructions. Biotinylated DM1 was added to CD47 antibody–streptavidin at a ratio of 3.5:1 to match a clinically available anti‐CD47‐DM1 drug‐to‐antibody ratio (DAR) of 3.5, and the mixture was incubated at room temperature for 30 min. Anti‐CD47‐DM1 was purified using a NAb Protein G spin column (89953, Thermo Scientific) and resuspended in PBS for functional experiments. The purity and drug–antibody ratio (DAR) was analyzed using NanoDrop 2000 (Thermo Fisher Scientific, USA), and DAR was calculated following the published method:^[^ ^44^ ^]^ (*ε*_mAb_^252^‐*Rε*_mAb_^280^)/(*Rε*_Drug_^280^‐*ε*_Drug_^252^), where *R* = *A*_252_/*A*_280_ = absorbance ratio, *ε*_mAb_^252^ = 9.41 ×10^4^ M^−1^ cm^−1^, *ε*_mAb_^280^ = 2.34 × 10^5^ M^−1^ cm^−1^, *ε*_DM1_^252^ = 2.64 × 10^5^ M^−1^ cm^−1^, *ε*_DM1_^280^ = 5.23 × 10^3^ M^−1^ cm^−1^. The integrity of ADC was confirmed with SDS‐PAGE.

### Statistical Analysis

All data are reported as mean ± standard deviation (s.d.). Statistical analysis was performed by GraphPad Prism 8. Two‐tailed unpaired t‐test was used for two‐group comparisons, and one‐way analysis of variance (ANOVA) was used for multiple comparisons. The survival benefit was determined using the two‐sided log‐rank test. The threshold for statistical significance was *p* < 0.05.

## Conflict of Interest

The authors declare no conflict of interest.

## Author Contributions

Z.T. and X.W. contributed equally to this work. C.L., P.T., Z. T., and X.W. conceived the project, designed all the experiments, analyzed the data, and wrote the manuscript. Z.T., M.T., J.W., J.Z., X. L., F.G., Y.F., and P.T. conducted the experiments and analyzed the data. All authors edited the manuscript.

## Supporting information

Supporting Information

Supplemental Movie 1

Supplemental Movie 2

Supplemental Movie 3

Supplemental Movie 4

Supplemental Movie 5

## Data Availability

The data that support the findings of this study are available in the supplementary material of this article.
