# Supplementary material for: Overcoming the On‐Target Toxicity in Antibody‐Mediated Therapies via an Indirect Active Targeting Strategy
Source: Adv Sci (Weinh). 2023 Jan 22;10(9):2206912. doi: 10.1002/advs.202206912 (PMC10037698; doi:10.1002/advs.202206912)
Supplement: Supplementary file 1 — Supporting Information [file ADVS-10-2206912-s004.pdf]

Supplementary Information

Overcoming the on-target toxicity in antibody-mediated therapies via an indirect active targeting strategy

Zhongjie Tang[1#], Xiaoyou Wang[1#], Mei Tang[1], Jin Wu[2], Jiexuan Zhang[1], Xinlong Liu[1], Feiyan Gao[1], Yu Fu[1], Peng Tang[2], Chong Li[1*]*

# These authors contributed equally to this work.
* Corresponding author. Email: chongli@swu.edu.cn; tp1232000@sina.com

Supplementary Materials list

Figure S1. Quantitative analysis of CD47 expression on different cells.
Figure S2. Quantification of total phospholipid content in CM.
Figure S3. Quantitative counting of different nanoparticles and efficiency of fluorescein-labeling.
Figure S4. Characterization of CM vesicles, NP, PCM@NP, and Anti-CD47-PCM@NP.
Figure S5. Transmission electron micrographs of NP, PCM@NP, and Anti-CD47-PCM@NP.
Figure S6. Determination of antibody loading on the surface of Anti-CD47-PCM@NP.
Figure S7. The binding affinity of antibody with CM measured by surface plasmon resonance.
Figure S8. Representative fluorescent images of uptake of NP, PCM@NP, and Anti-CD47-PCM@NP by RAW264.7 macrophages and 4T1 cells.
Figure S9. Analysis of the expression level of CD47 in CD47$^{-/-}$ 4T1 cells.
Figure S10. Appearance of the Real-time Single cell Multi-modal Analyzer.
Figure S11. *In vivo* and *ex vivo* targeting ability of NP and PCM@NP in tumor-bearing mice models.
Figure S12. Representative fluorescent images in tumor, heart, lung, liver, spleen and kidney slices after different treatments.
Figure S13. Pharmacokinetic analysis of Anti-CD47-PCM@NP.
Figure S14. Representative images and phagocytic index of C57BL/6 bone marrow-derived macrophages phagocytosing tumor cells after treatment.

Figure S15. Ki67 percentage and immune cell infiltration in tumor sections after Anti-CD47-PCM@NP treatment, by immunohistochemistry and immunofluorescence staining.
Figure S16. Expression of immune signatures for the identification of tumor-infiltrating immune cell.
Figure S17. Construction of HuNSG mouse model bearing PDX tumor.
Figure S18. Anti-tumor efficacy study on HuNSG PDX tumor-bearing mice.
Figure S19. Body weight curves during the Anti-CD47-PCM@NP treatment.
Figure S20. H&E staining of the main organs in mice treated with Saline, PCM@NP, Anti-CD47, Anti-CD47-PCM@NP.
Figure S21. *C. albicans* infection status in mice kidney after different treatments.
Figure S22. Characterization of NP/PTX, PCM@NP/PTX, and Anti-CD47-PCM@NP/PTX.
Figure S23. The INTACT strategy enabled the co-delivery of antibody with small-molecule chemotherapeutic agent for combinational therapy with precise targeting.
Figure S24. Ki67 percentage and immune cell infiltration in tumor sections after Anti-CD47-PCM@NP/PTX treatment, by immunohistochemistry and immunofluorescence staining.
Figure S25. Ki67 percentage and immune cell infiltration in tumor sections after ADC-PCM@NP treatment, by immunohistochemistry and immunofluorescence staining.
Figure S26. Body weight curves during the ADC-PCM@NP treatment.
Figure S27. H&E staining of the main organs in mice treated with Saline, ADC and ADC-PCM@NP.
Figure S28. Characterization of CMV-Anti-CD47-Lip and functional analysis of the indirect active targeting *in vitro*.
Figure S29. Flow cytometric analysis of the HER2 expression on the surface of TUBO and 4T1 cells.
Figure S30. AST, CTN-I, LDH indexes, and H&E staining of hearts and lungs of mice after Anti-HER2-PCM@NP treatments.
Table S1. Characterization of the CM vesicles, PLGA NPs and PLGA NPs loaded with PTX.
Table S2. Antibody and metal isotopes for CyTOF.
Table S3. The expression of specific markers of each cluster.
Table S4. Hematotoxicity analysis of Anti-CD47.
Table S5. *In vitro* anticancer activity test of Anti-CD47-PCM@NP/PTX and other formulations.
Table S6. Hematotoxicity analysis of ADC.
Movie S1. Instrumental field of view for single cell fluorescence signal detection by the Real-time Single cell Multi-modal Analyzer nanoprobe.
Movie S2. Microscopic field of view for single cell fluorescence signal detection by the Real-time Single cell Multi-modal Analyzer nanoprobe.
Movie S3. The binding of free Anti-CD47 to the target cells adsorbed in the cavity of the microfluidic chip in the flowing state.

Movie S4. *In vitro* transfer of CD47 antibody from Anti-CD47-PCM@NP to the surface of 4T1 cells was assessed by microfluidic assay.

Movie S5. No significant surface binding of Anti-CD47 or PCM@NP internalization was observed on CD47$^{-/-}$ 4T1 cells.

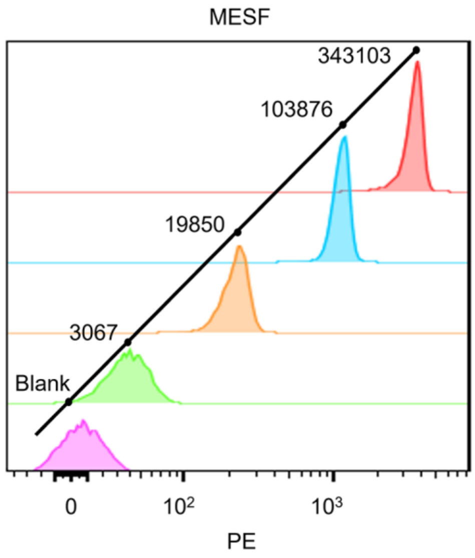

Cell	CD47 protein expression (molecules per cell)
RBC (Red blood cells)	13180±3280
PLT (Platelets)	33556±5250
4T1 (Mouse breast cancer cell line)	344056±21945
RM-1 (Mouse prostate cancer cell line)	538735±47255
A20 (Mouse B-cell lymphoma cell line)	170033±18652
P388 (Mouse leukemia cells)	171817±16344
AKR (Mouse esophageal carcinoma cell line)	163509±20654
MCF-7 (Human breast cancer cell line)	153475±37662
MDA-MB-231 (Human breast cancer cell line)	369907±23593
MDA-MB-468 (Human breast cancer cell line)	380990±23981
HPAC (Human pancreatic cancer cell line)	197865±17889
HCT116 (Human colon cancer cell line)	210863±32980
A549 (Human non-small cell lung cancer cell line)	144966±29856
NCI-N87 (Human gastric cancer cell line)	52491±2788
TE-1 (Human esophageal cell line)	138890±9076
OVCAR-3 (Human ovarian cancer cell line)	798145±82092
HepG2 (Human hepatoma cell line)	63223±4819

Figure S1. Standard curve by Quantum PE MESF microbeads and quantitative analysis of CD47 expression on different cells by flow cytometry. Data are shown as means ± SD (n = 3).

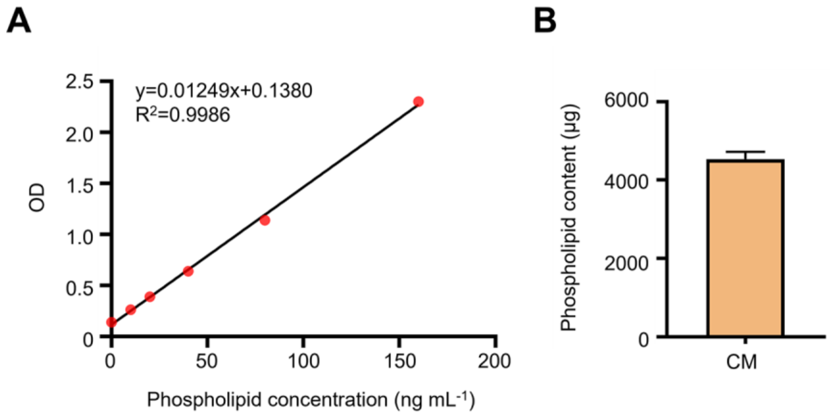

Figure S2. A, The standard curve of phospholipid by ELISA. **B,** The phospholipid content of CM. Data were presented as mean ± SD (n = 6).

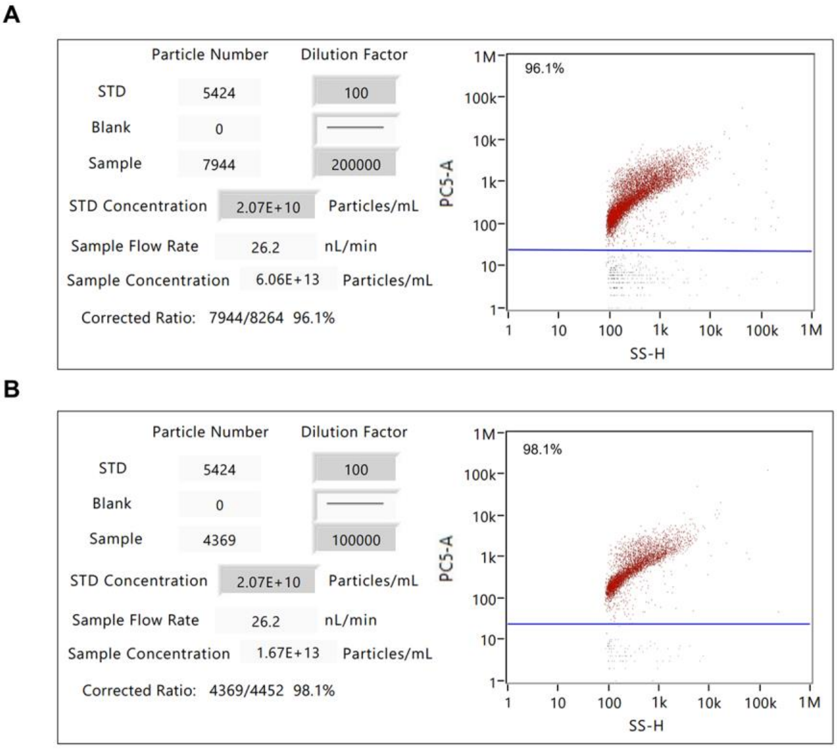

Figure S3. Quantitative counting of different nanoparticles and efficiency of fluorescein-labeling by Flow NanoAnalyzer. **A,** DiD-labeled liposomes (Lip/DiD), and **B,** Anti-CD47-PCM@NP/DiD. The instrument accuracy was validated by the data of standardized 120 nm Lip/DiD (6.06×10^{13} particles per mL, consistent with previous reports[1]).

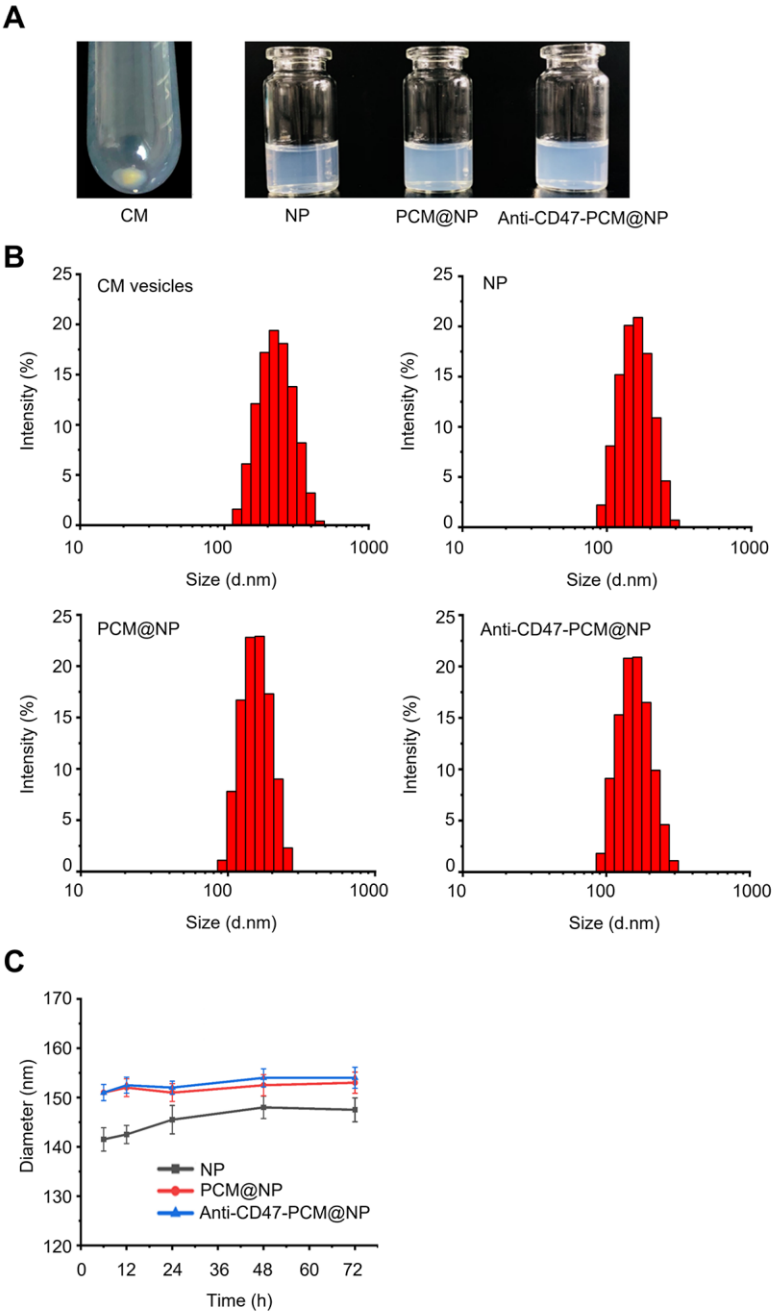

Figure S4. A, Appearance of CM extracted from 4T1 cells, NP, PCM@NP and Anti-CD47-PCM@NP**. B,** The size distribution of CM vesicles, NP, PCM@NP, and Anti-CD47-PCM@NP**. C,** *In vitro* stability of NP, PCM@NP and Anti-CD47-PCM@NP in saline at 37 °C for 72 h. Data represented as mean ± SD (n = 3).

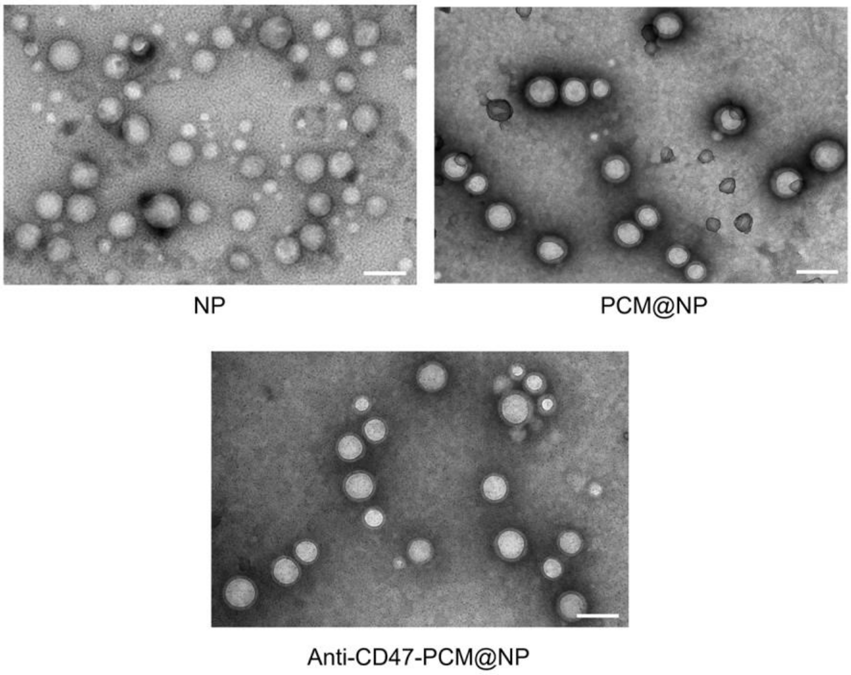

Figure S5. Transmission electron micrographs of NP, PCM@NP, and Anti-CD47-PCM@NP, scale bar = 200 nm.

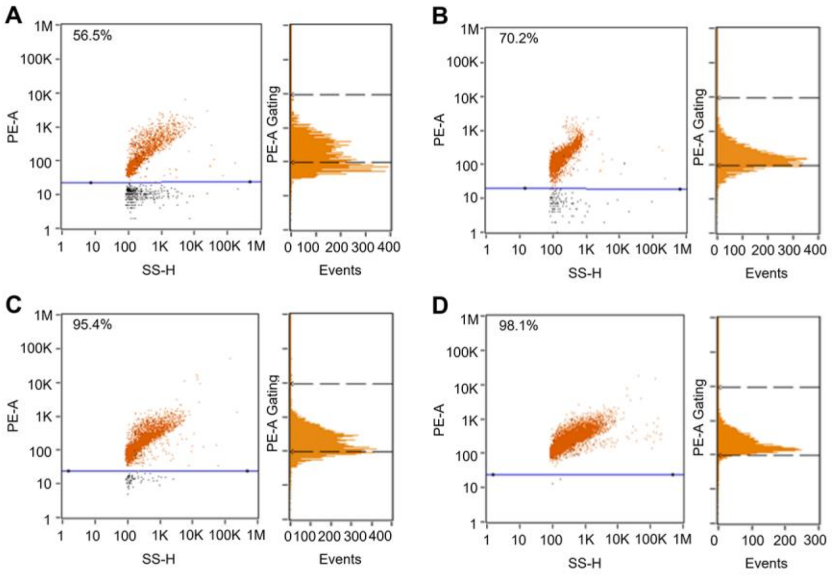

Figure S6. Determination of antibody loading on the surface of Anti-CD47-PCM@NP by Flow NanoAnalyzer. (Antibody concentration: (**A**) 10 μg mL^{-1}, (**B**) 20 μg mL^{-1}, (**C**) 30 μg mL^{-1}, and (**D**) 40 μg mL^{-1}). The fluorescence labeling rate increased with the increase of the antibody concentration, 40 μg mL^{-1} antibody provided a 98.1 % fluorescent labeling rate of the formulation after ultracentrifugation.

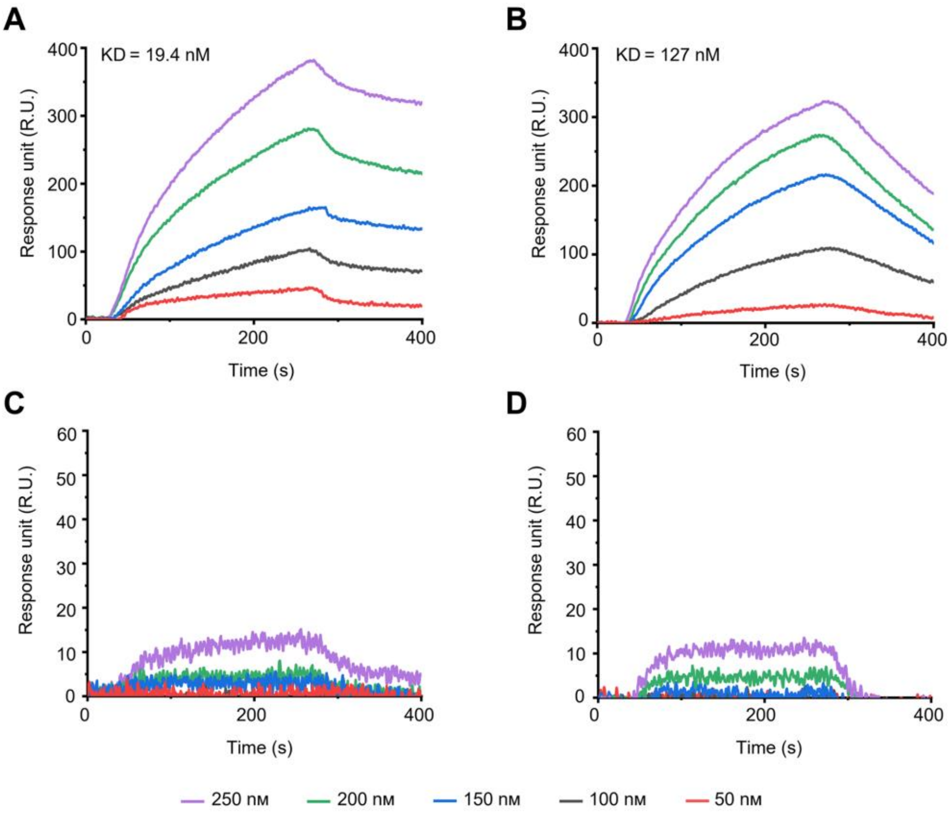

Figure S7. The binding affinity between **A,** N-hydroxysuccinimide palmitate modified CD47 antibodies and CM vesicles, **B,** Anti-CD47 and CM vesicles, **C,** IgG and CM vesicles, **D,** Anti-CD47 and Lip measured by surface plasmon resonance (SPR).

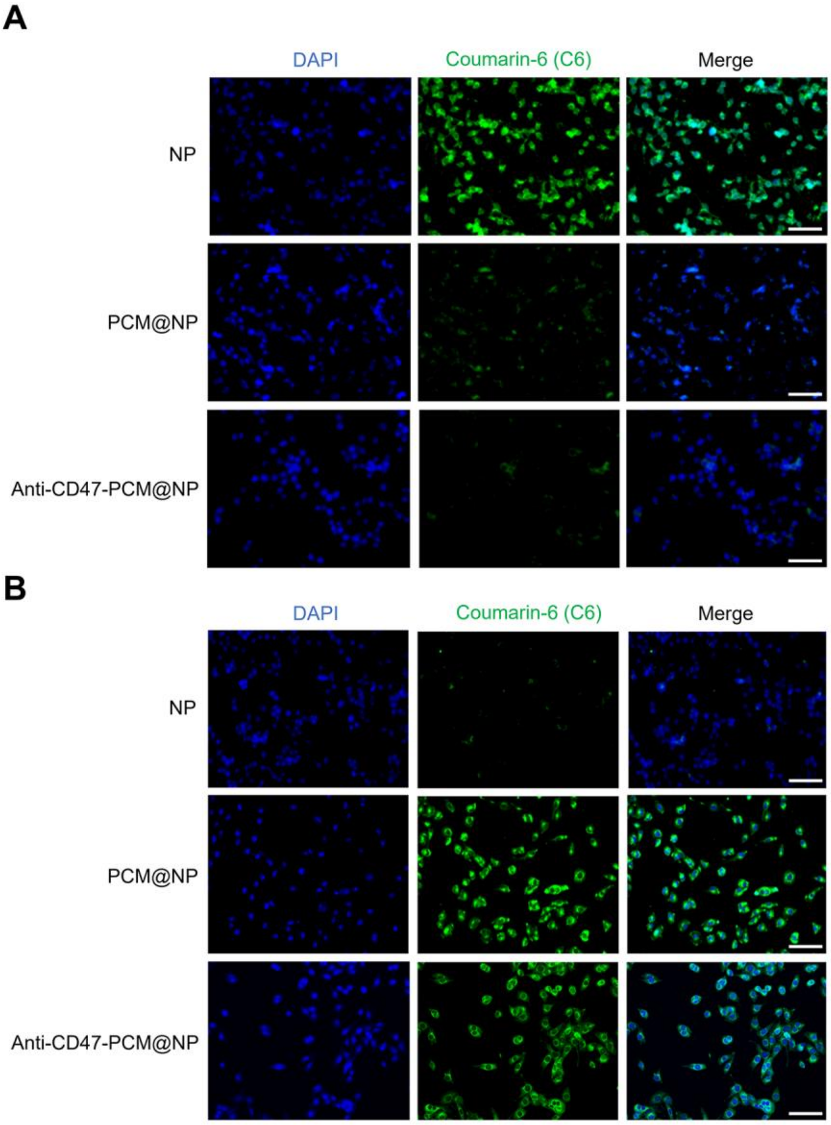

Figure S8. Uptake of NP, PCM@NP, and Anti-CD47-PCM@NP by (**A**) RAW264.7 macrophages and (**B**) 4T1 cells. Formulations were labeled with coumarin-6 (C6), scale bar = 100 μm.

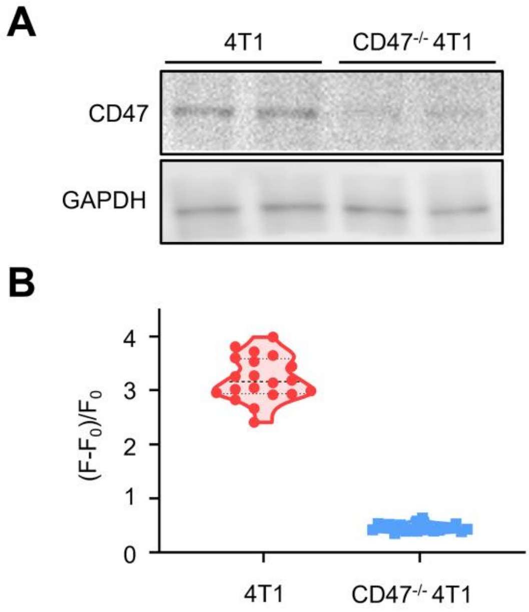

Cell	CD47 protein expression (molecules per cell)
4T1	344056±21945
$CD47^{-/-}$4T1	99835±14305

Figure S9. A, CD47 protein levels of $CD47^{-/-}$ 4T1 cells analyzed by western blotting. The $CD47^{-/-}$ 4T1 cells were produced by treating 4T1 cells with siCD47 for 48 hours. The siRNA concentration was 100 nM. GAPDH, glyceraldehyde-3-phosphate dehydrogenase. **B,** The expression of CD47 protein on the surface of 4T1 cells and $CD47^{-/-}$ 4T1 cells measured by the Real-time Single cell Multi-modal Analyzer (F: fluorescence intensity of cell membrane surface, F_0: background fluorescence intensity of culture dish, n = 20) and the flow cytometry analysis (standard curve constructed by Quantum PE MESF microbeads).

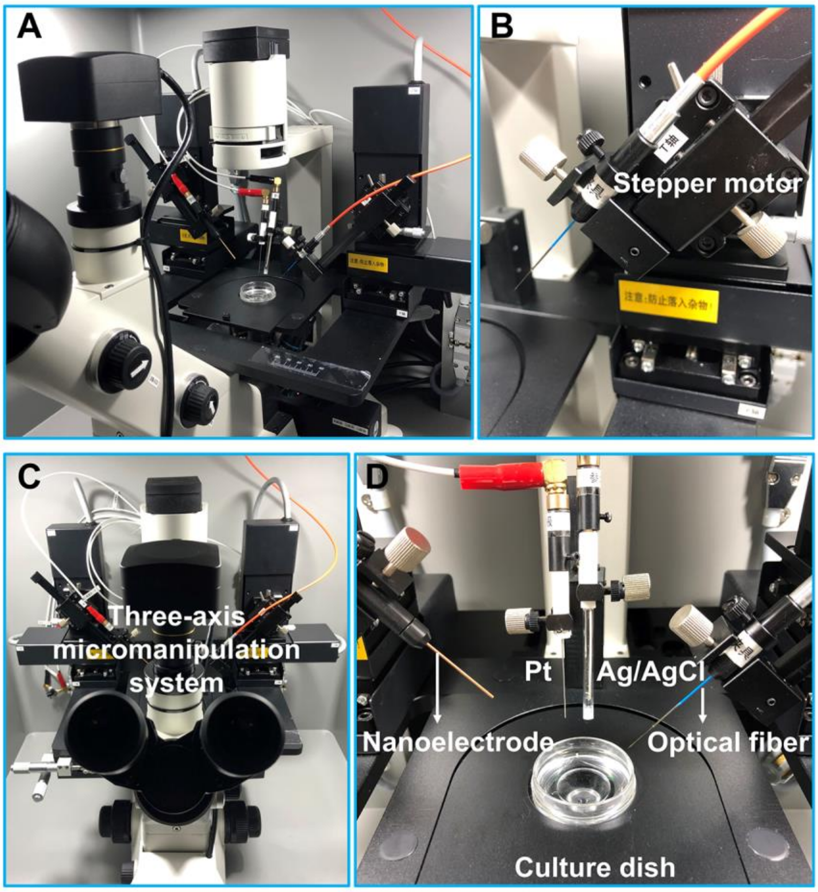

Figure S10. A, Appearance of the Real-time Single cell Multi-modal Analyzer, the detection system consists of optical micro/nanofibers and nanoelectrodes. **B-D,** Enlarged view of the three-axis micromanipulation system equipped with a stepper motor to precisely control the movement of micro/nano optical fiber and nanoelectrode.

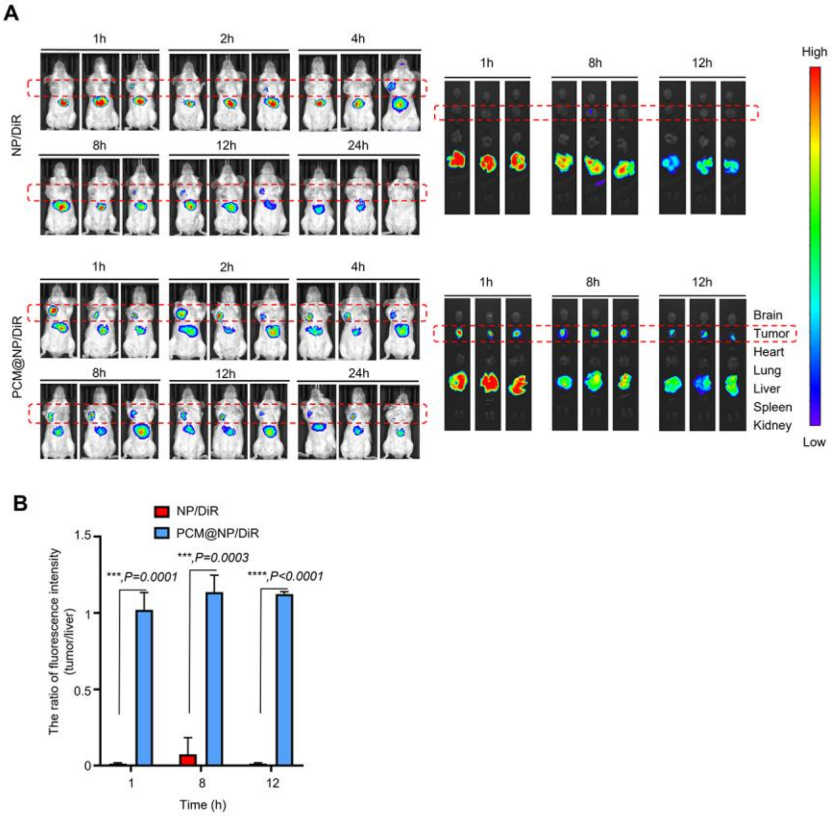

Figure S11. A, *In vivo* and *ex vivo* targeting ability of NP and PCM@NP in tumor-bearing mice models determined by live imaging. **B,** The semi-quantitative analysis of the ratio of fluorescence intensity (tumor/liver) of *ex vivo* imaging. Data represented as mean ± SD (n = 3) (*p<0.05, **p<0.01, ***p<0.001, ****p<0.0001; NS represents non-significance).

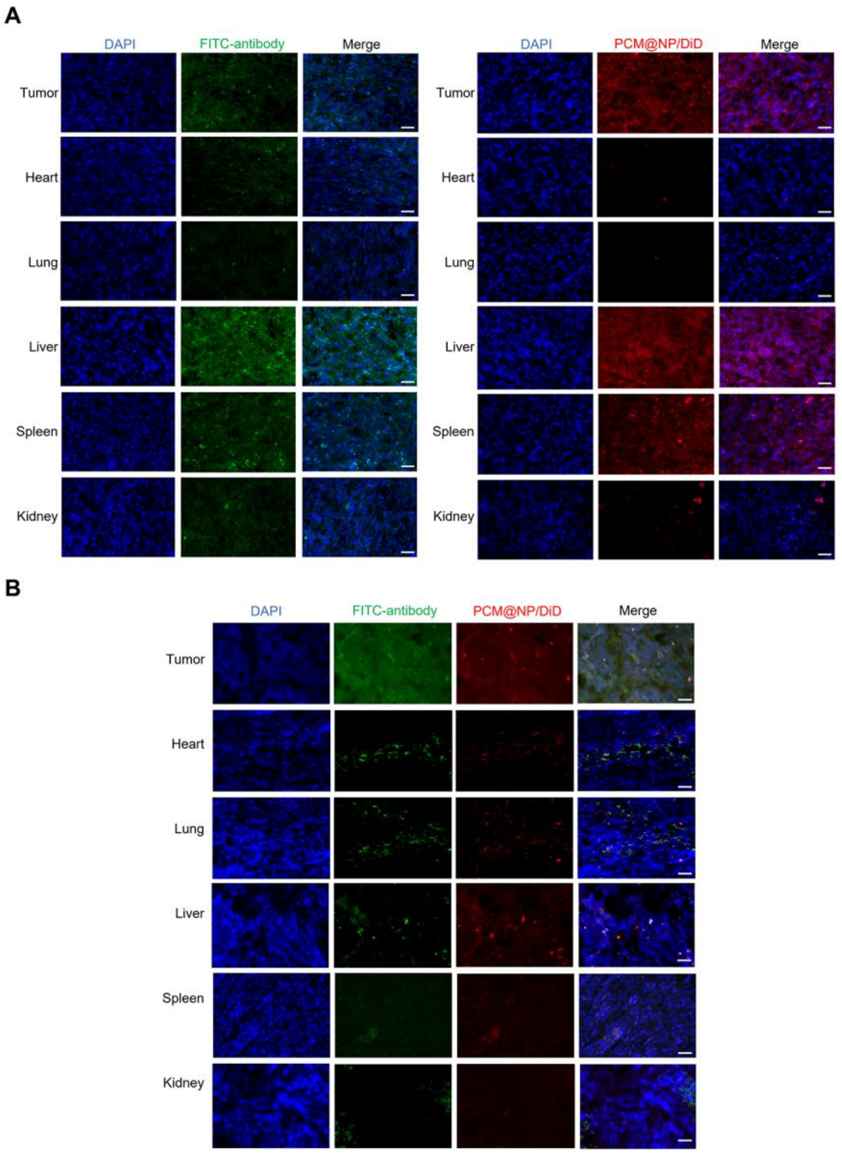

Figure S12. A, Representative fluorescent images in tumor, heart, lung, liver, spleen and kidney slices after treatment of FITC-antibody, PCM@NP/DiD, and **B,** FITC-Anti-CD47-PCM@NP/DiD. Scale bar = 50 μm.

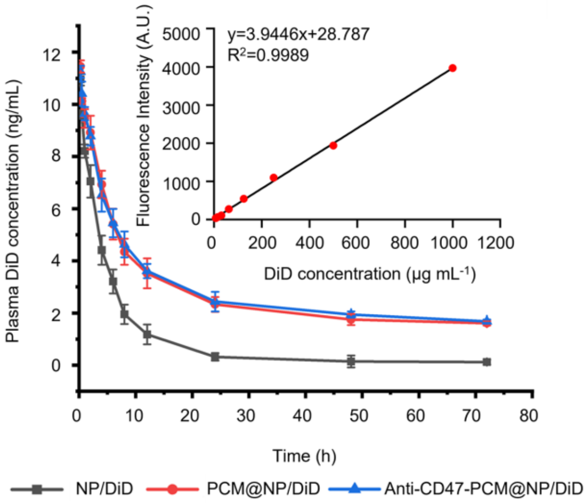

Figure S13. Plasma DiD concentration in SD rats after i.v. administration of NP/DiD, PCM@NP/DiD, and Anti-CD47-PCM@NP/DiD, respectively (n = 6), with standard curve of DiD in plasma.

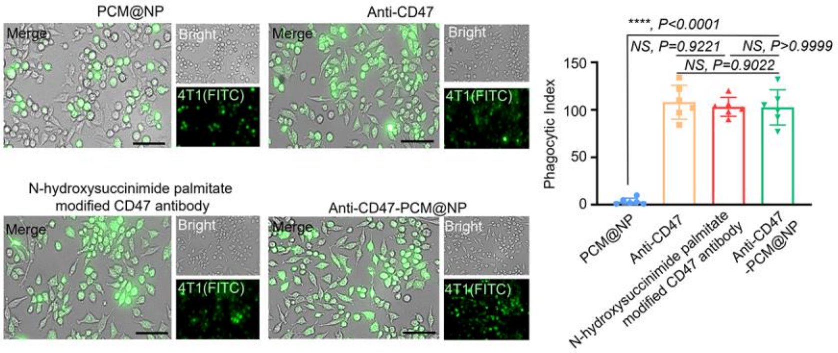

Figure S14. Representative images and phagocytic index of C57BL/6 bone marrow-derived macrophages (BMDM) phagocytosing tumor cells following treatment with PCM@NP, Anti-CD47, N-hydroxysuccinimide palmitate modified CD47 antibody, and Anti-CD47-PCM@NP. Scale bar = 50 μm. Data represented as mean ± SD (n = 6). (*p<0.05, **p<0.01, ***p<0.001, ****p<0.0001; NS represents non-significance).

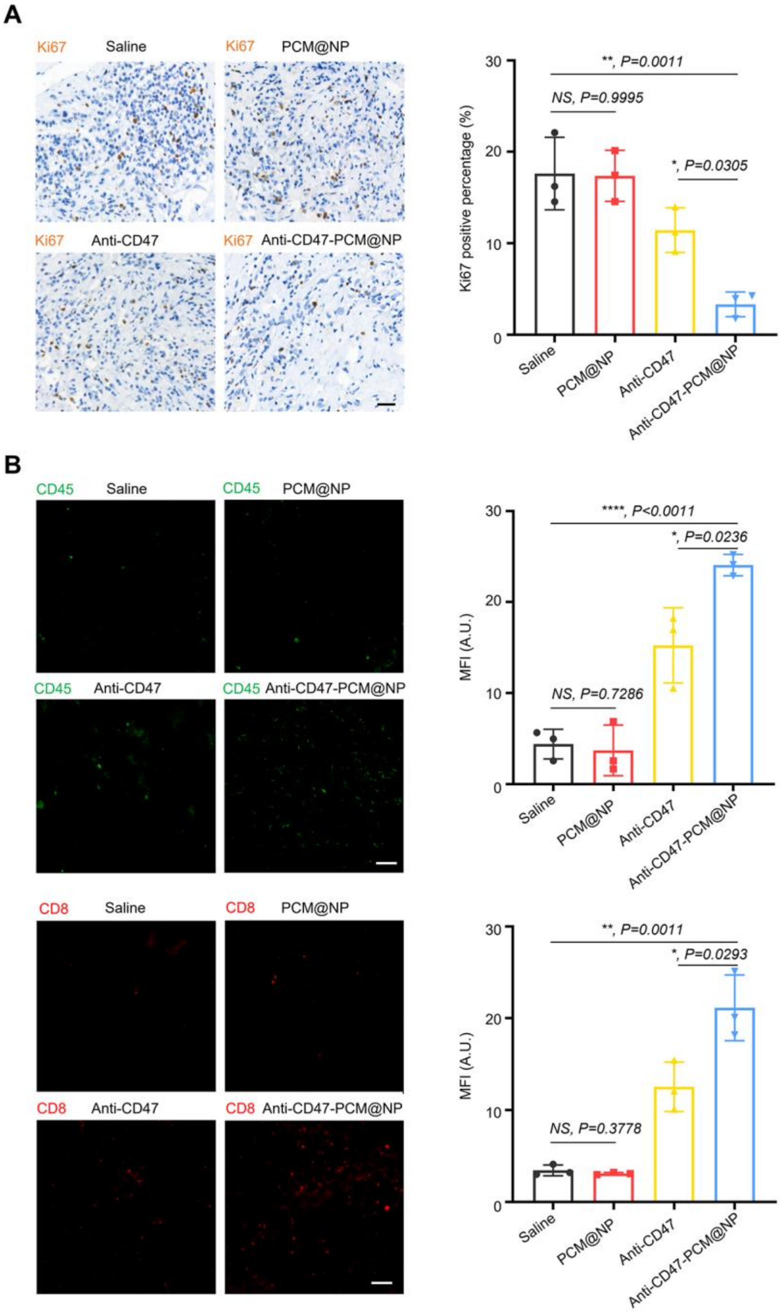

Figure S15. A, Immunohistochemical staining and positive percentage of Ki67 in tumor. Scale bar = 100 μm. **B,** Immunofluorescence staining of CD45 and CD8 in tumor tissue sections after treatment, and quantitative analyses of the mean fluorescence intensity. Scale bar = 100 μm. Data represented as mean ± SD (n = 3). (*p<0.05, **p<0.01, ***p<0.001, ****p<0.0001; NS represents non-significance).

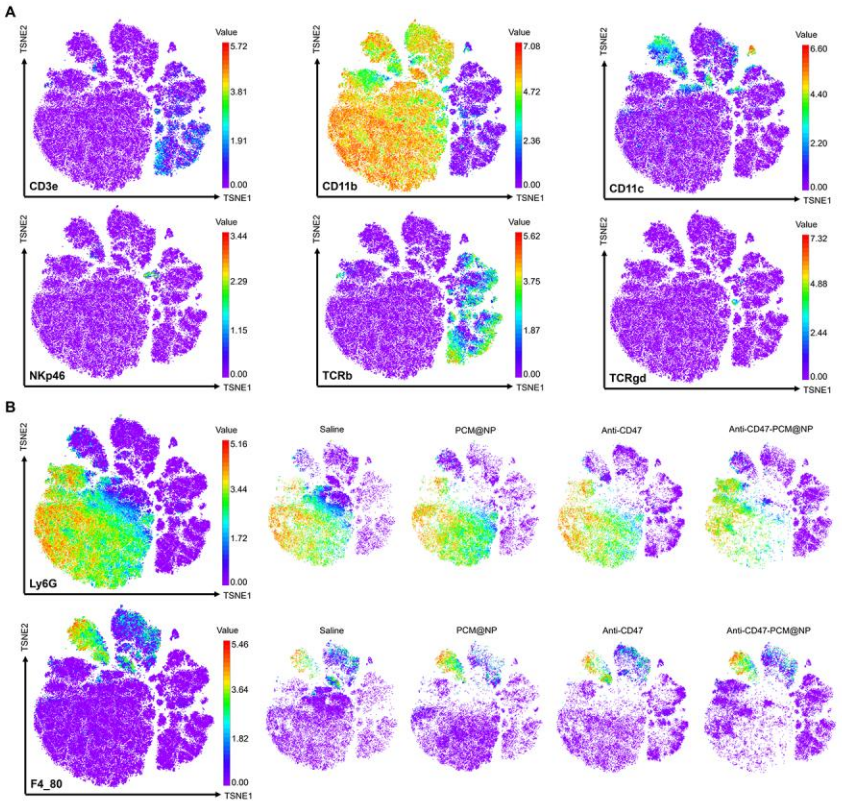

Figure S16. A, Expression of immune signatures for the identification of tumor-infiltrating immune cell, including CD3e, CD11b, CD11c, NKp46, TCRb, and TCRgd. **B,** tSNE visualization of all samples with the expression of Ly6G and F4_80 respectively.

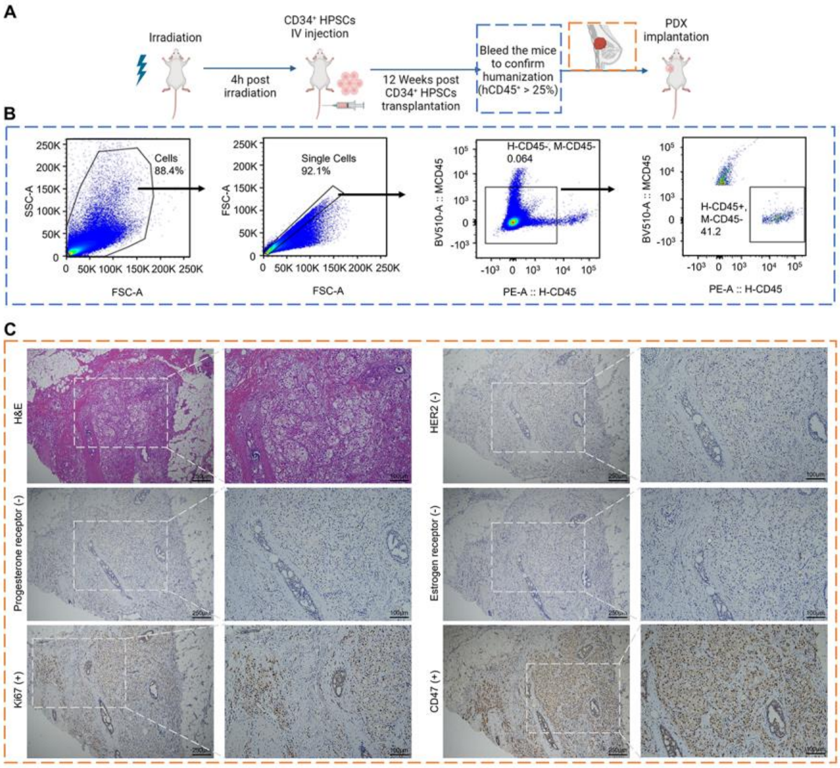

Figure S17. A, Experimental design for the generation of HuNSG mice bearing PDX tumor. **B,** The contents of human and mouse derived CD45$^+$ cells in mouse peripheral blood were detected by flow cytometry. **C,** Immunohistochemical section of tumor tissue from the clinical triple-negative breast cancer patient.

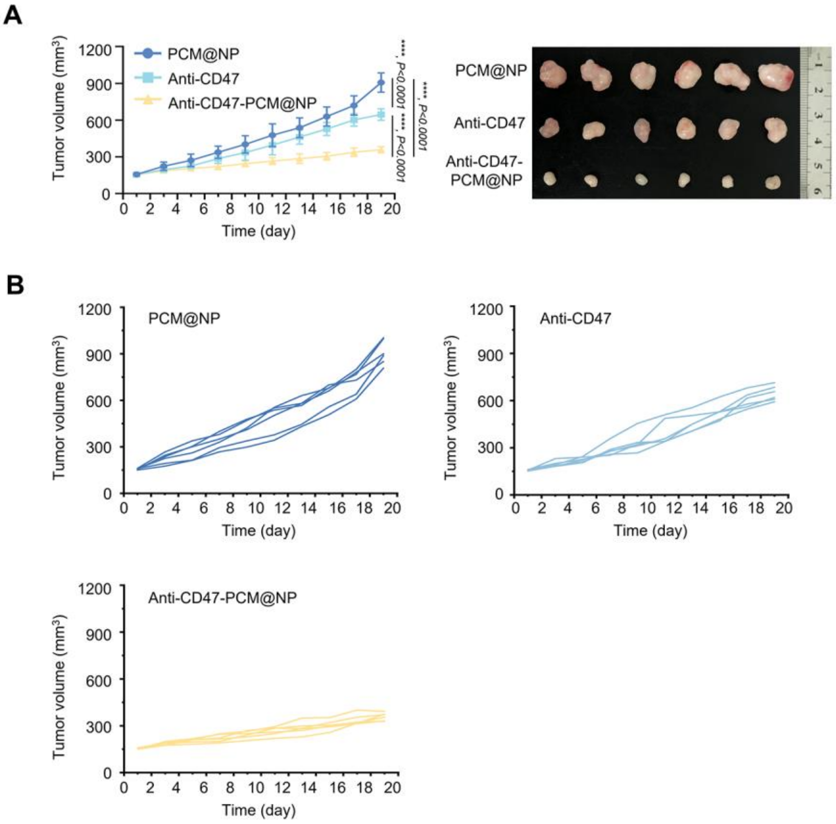

Figure S18. A, Growth curves and picture of the tumor tissues after treatment on HuNSG PDX tumor-bearing mice**. B,** Individual tumor volume growth curves in each group. Data represented as mean ± SD (n = 6). (*p<0.05, **p<0.01, ***p<0.001, ****p<0.0001; NS represents non-significance).

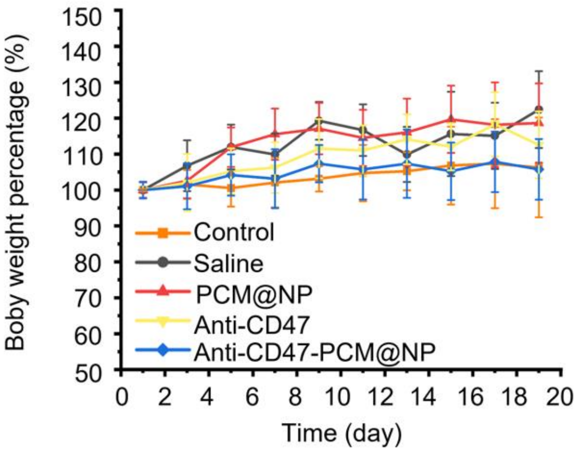

Figure S19. Body weight curves during the treatment of Anti-CD47-PCM@NP, untreated healthy mice were set as the control group. Data represented as mean ± SD (n = 6).

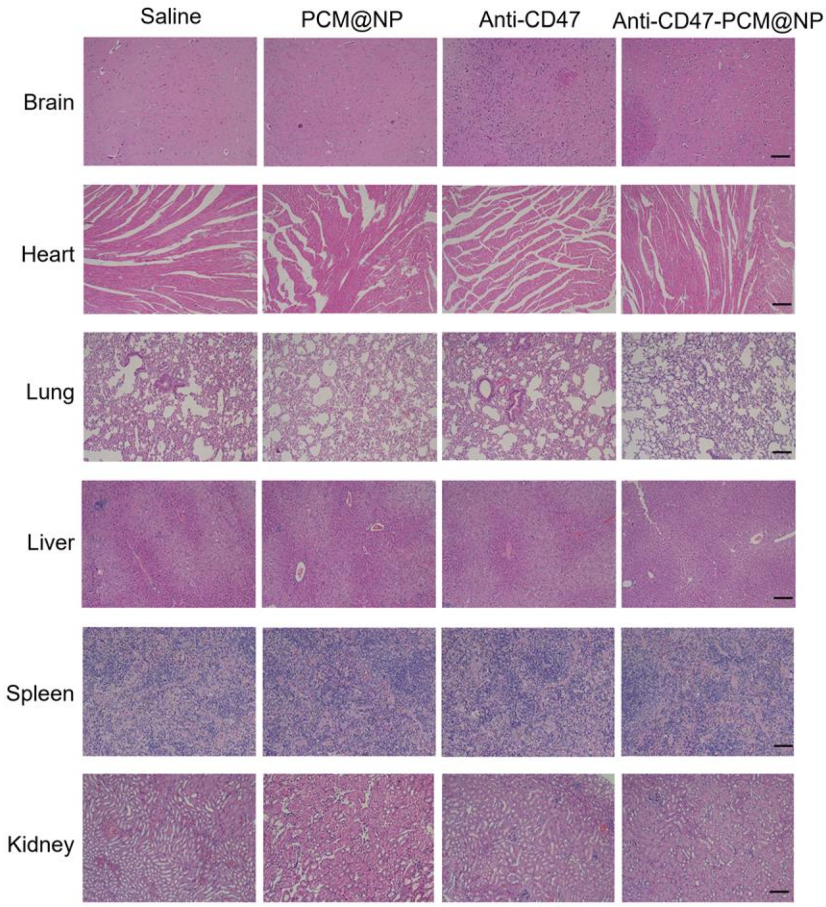

Figure S20. H&E staining of the main organs in mice with different treatments (Saline, PCM@NP, Anti-CD47, Anti-CD47-PCM@NP), scale bar = 200 μm.

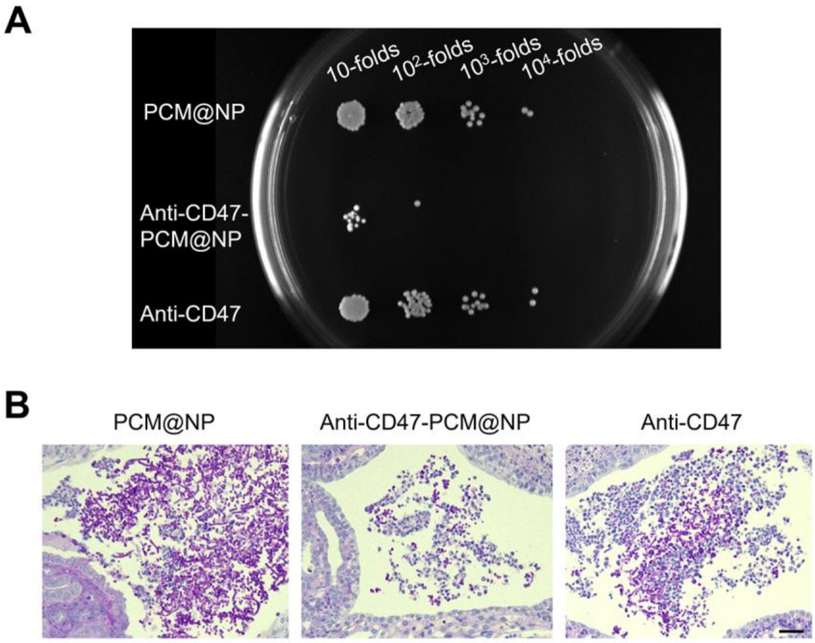

Figure S21. A, The tissue burden study of the PCM@NP, Anti-CD47-PCM@NP, and Anti-CD47 groups at day 7 after infection of *C. albicans*. **B,** Kidney sections and *C. albicans* infection status after different treatments stained with PAS (*C. albicans* is purple-red), scale bar = 100 μm.

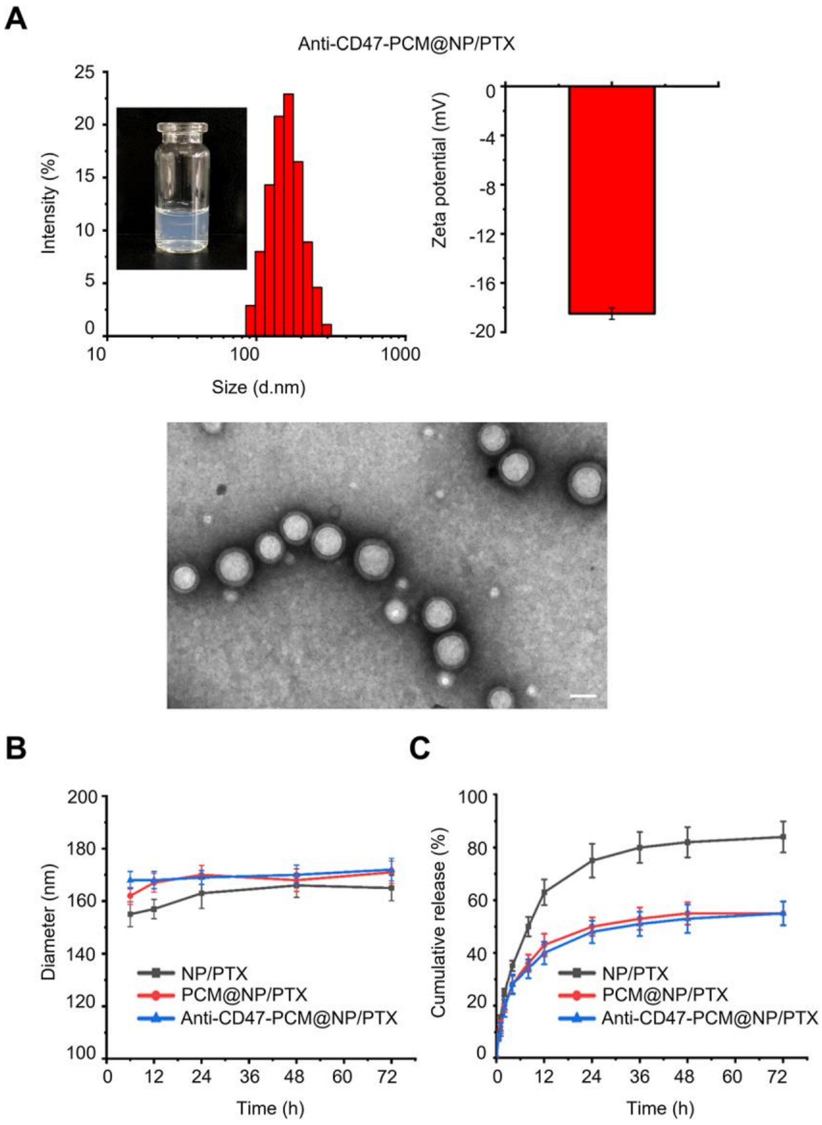

Figure S22. A, Appearance, size distribution, zeta potential and transmission electron micrographs of Anti-CD47-PCM@NP/PTX. Scale bar = 100 nm. **B,** *In vitro* stability of NP/PTX, PCM@NP/PTX, and Anti-CD47-PCM@NP/PTX in saline at 37 °C for 72 h. **C,** *In vitro* cumulative release profiles of PTX released from Anti-CD47-PCM@NP/PTX. Data represented as mean ± SD (n = 3).

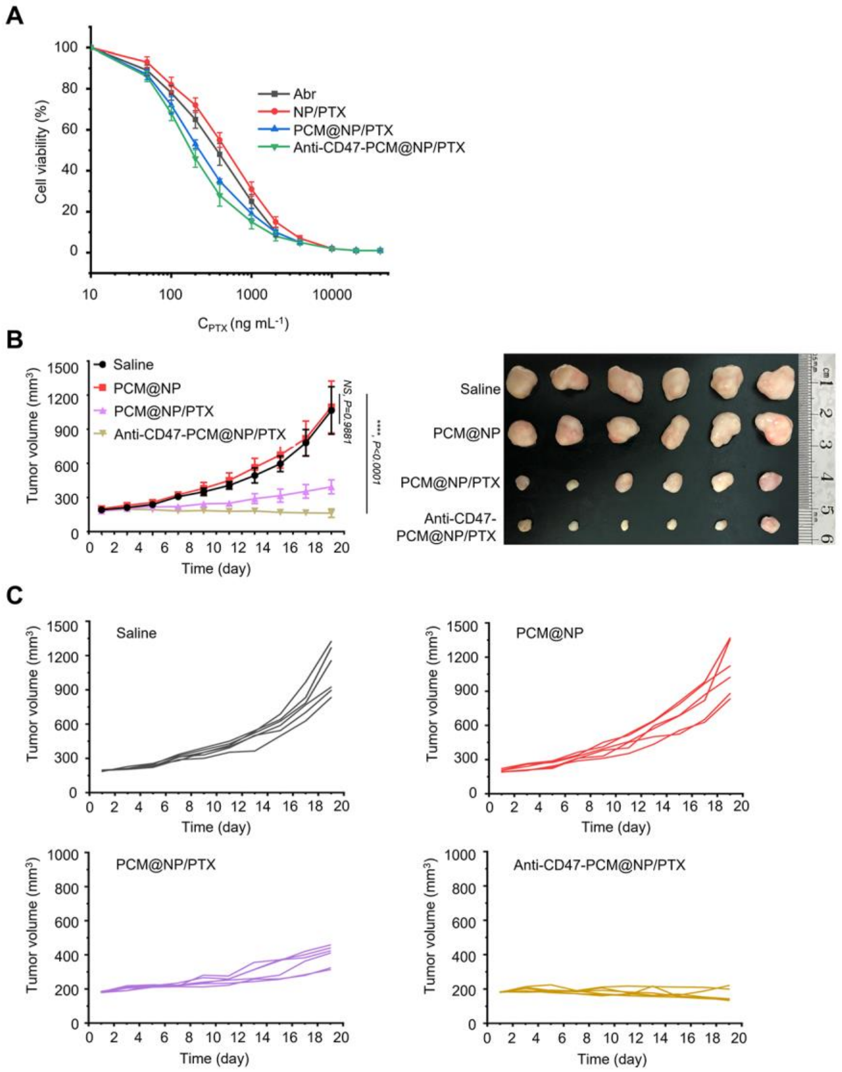

Figure S23. The INTACT strategy enabled the co-delivery of antibody with small-molecule chemotherapeutic agent for combinational therapy with precise targeting. **A,** *In vitro* anti-tumor activity test of Anti-CD47-PCM@NP/PTX (n = 6). **B,** Average tumor growth curves, picture of the tumor tissues after the treatment. **C,** Individual tumor growth curves in each group. Data represented as mean ± SD (n = 6). (*p<0.05, **p<0.01, ***p<0.001, ****p<0.0001; NS represents non-significance).

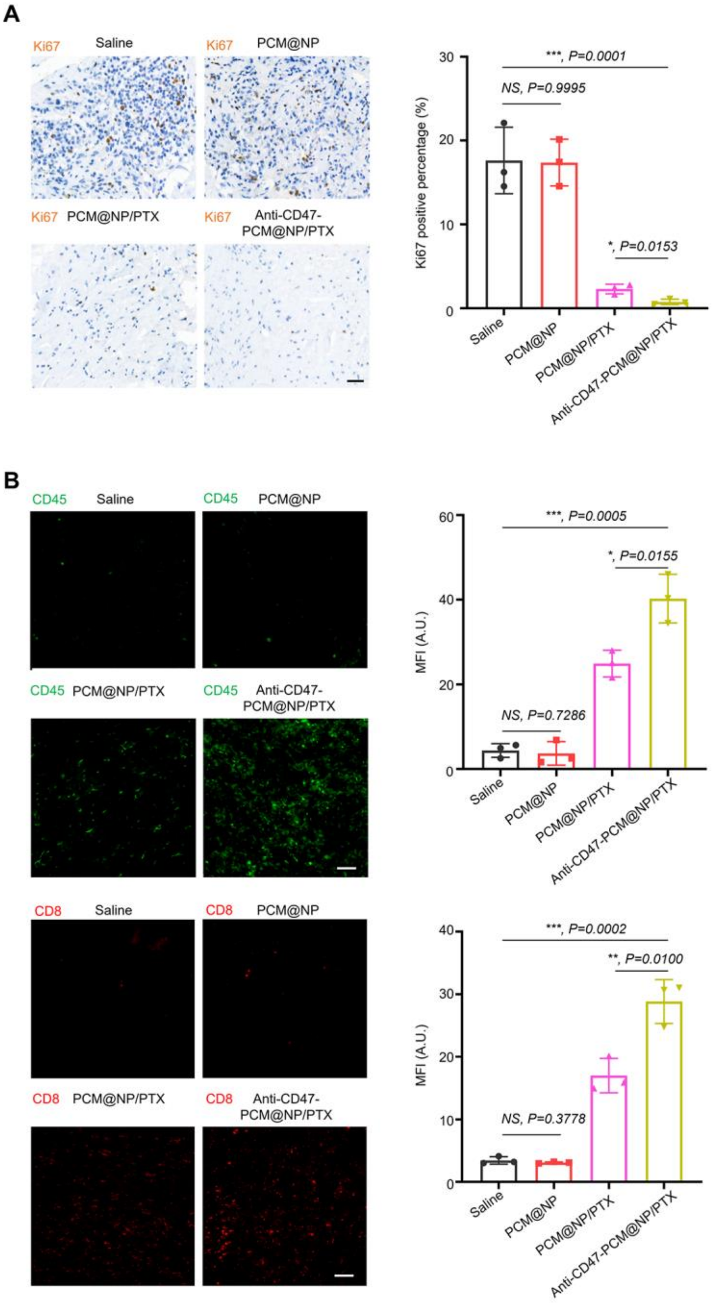

Figure S24. A, Immunohistochemical staining and positive percentage of Ki67 in tumor. Scale bar = 100 μm. **B,** Immunofluorescence staining of CD45 and CD8 in tumor tissue sections after treatment, and quantitative analyses of the mean fluorescence intensity. Scale bar = 100 μm. Data represented as mean ± SD (n = 3). (*p<0.05, **p<0.01, ***p<0.001, ****p<0.0001; NS represents non-significance).

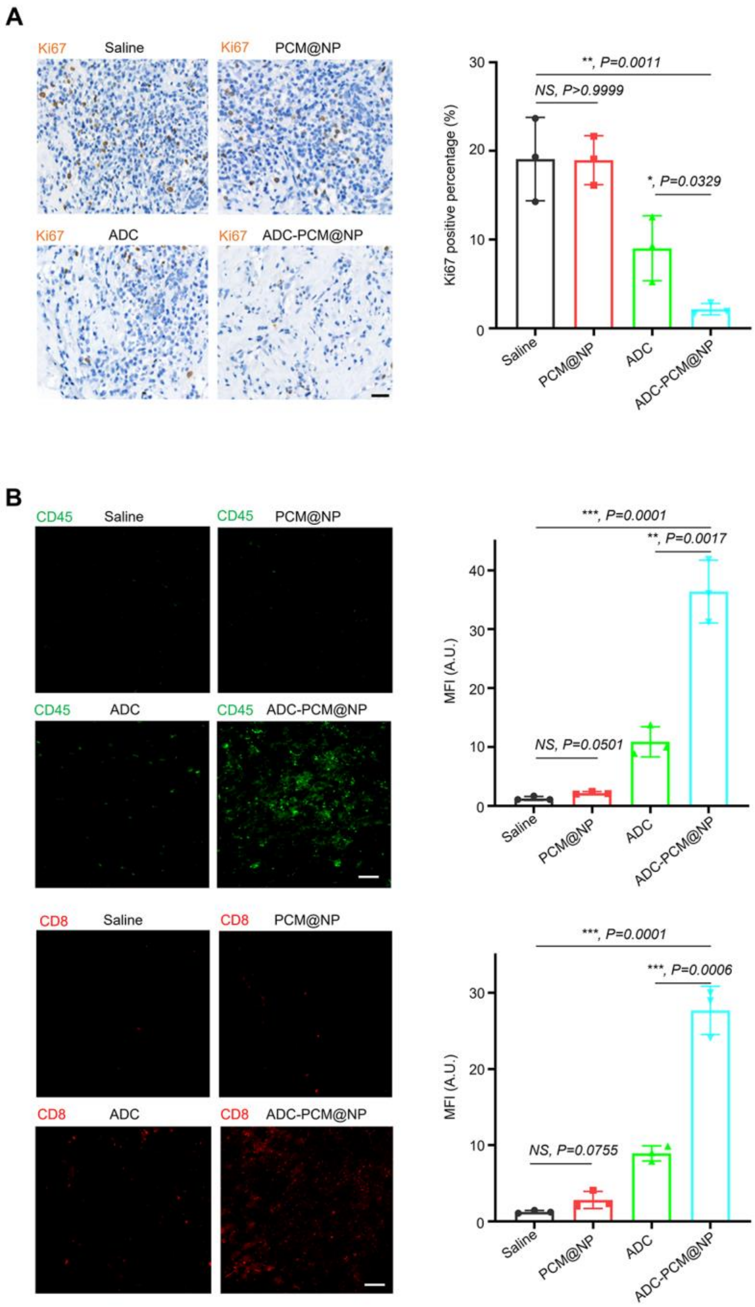

Figure S25. A, Immunohistochemical staining and positive percentage of Ki67 in tumor. Scale bar =100 μm. **B,** Immunofluorescence staining of CD45 and CD8 in tumor tissue sections after treatment, and quantitative analyses of the mean fluorescence intensity. Scale bar = 100 μm. Data represented as mean ± SD (n = 3). (*p<0.05, **p<0.01, ***p<0.001, ****p<0.0001; NS represents non-significance).

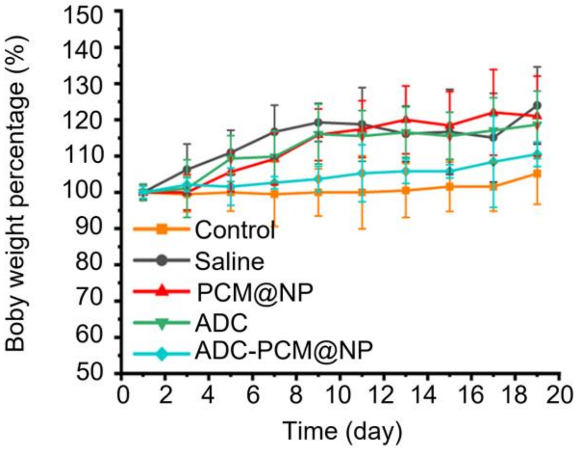

Figure S26. Body weight curves during the treatment of ADC-PCM@NP，untreated healthy mice were set as the control group. Data represented as mean ± SD (n = 6).

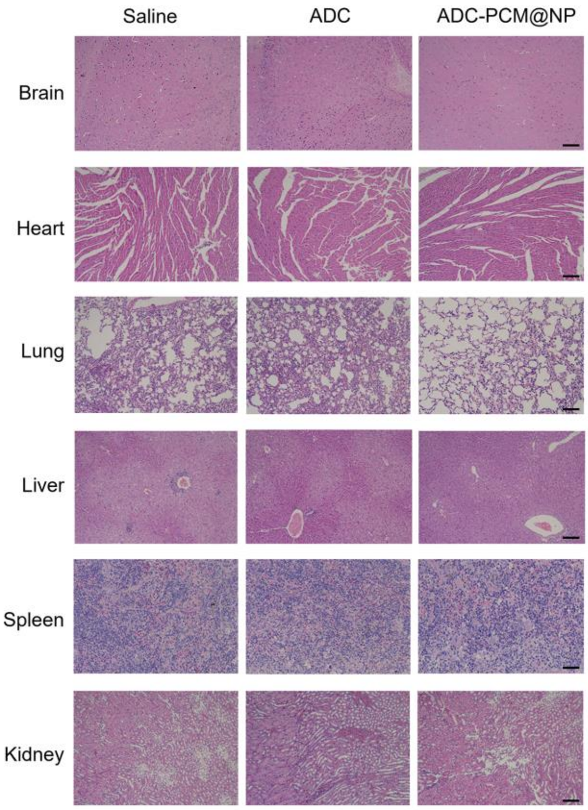

Figure S27. H&E staining of the main organs in mice with different treatments (Saline, ADC and ADC-PCM@NP), scale bar = 200 μm.

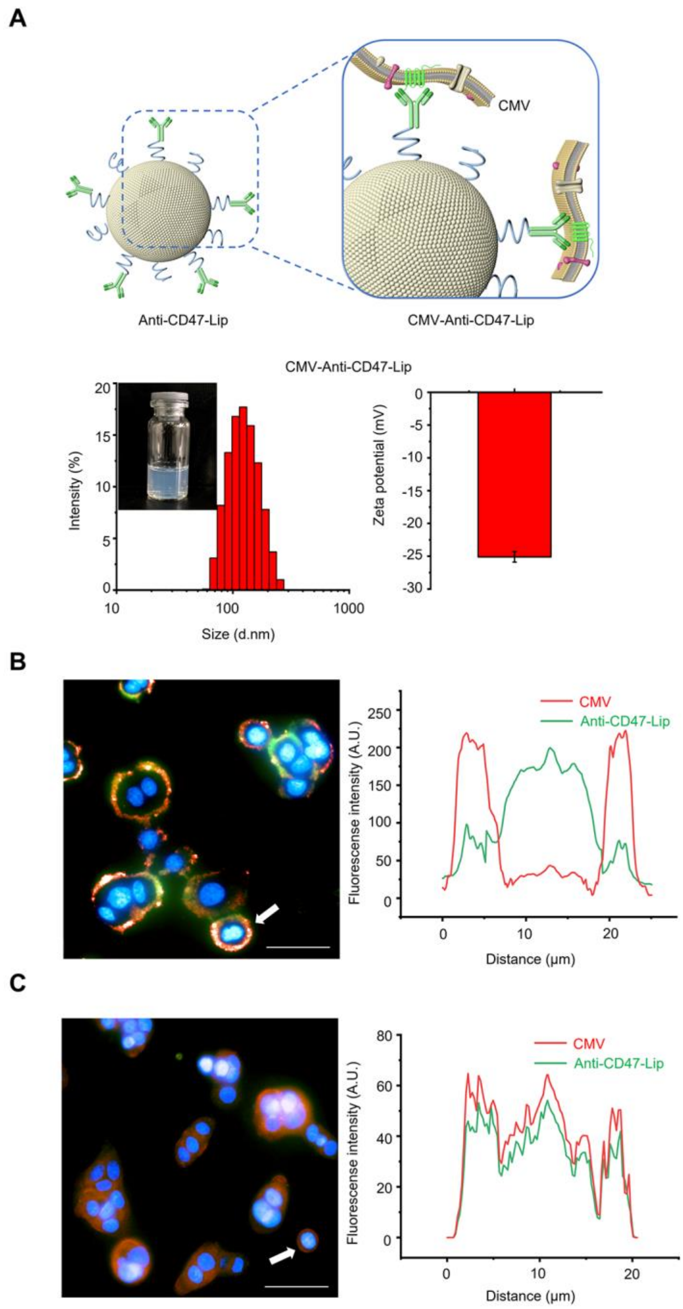

Figure S28. A, Schematic diagram, appearance, size distribution, and zeta potential of CMV-Anti-CD47-Lip. Data represented as mean ± SD (n = 3). **B,** Colocalization and distribution map of CMV (red) and Anti-CD47-Lip (green) on the surface of 4T1 cells highly expressing CD47 by CLSM. Scale bar = 30 μm. **C,** Colocalization and distribution map of CMV (red) and Anti-CD47-Lip (green) on the surface of $CD47^{-/-}$ 4T1 cells with low expression of CD47 by CLSM. Scale bar = 30 μm.

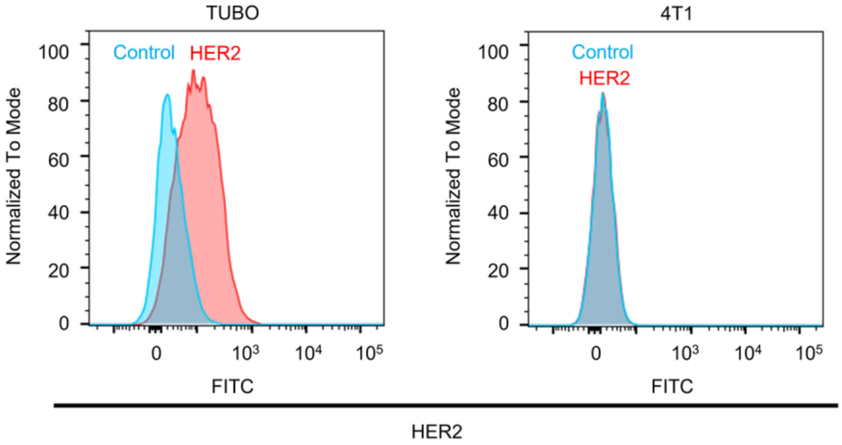

Figure S29. Flow cytometric analysis of the HER2 expression on the surface of TUBO and 4T1 cells.

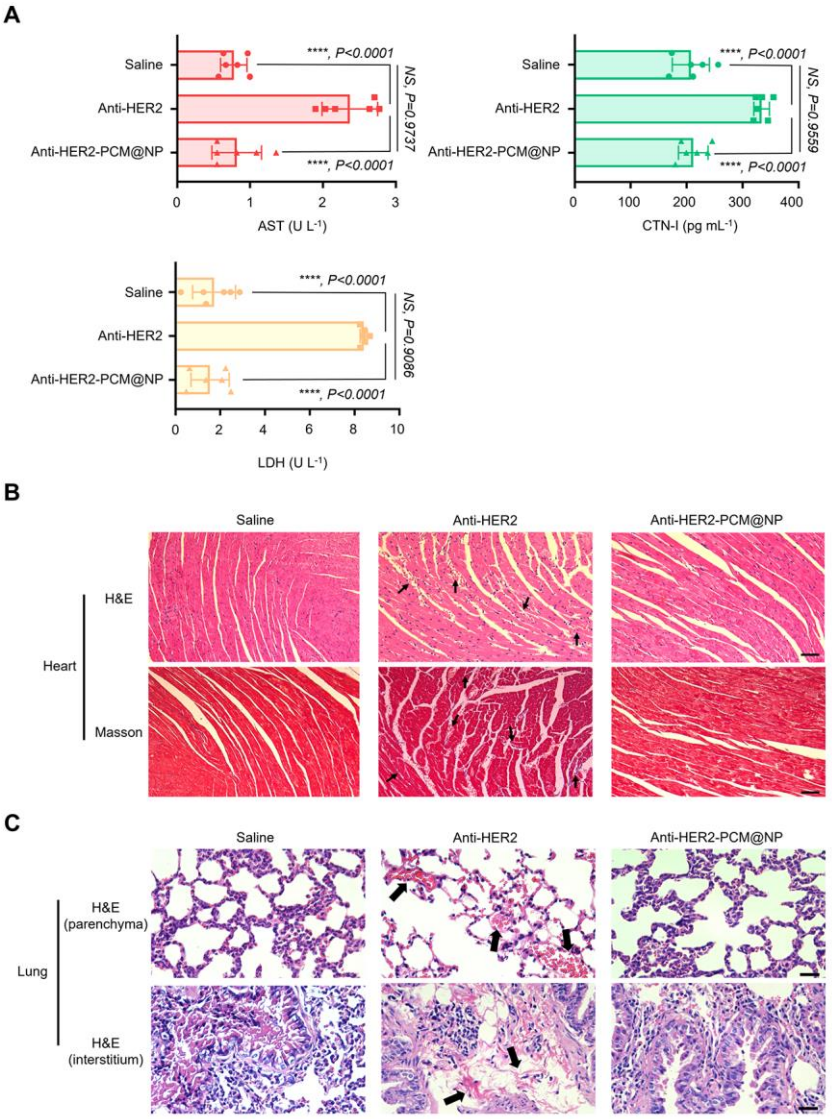

Figure S30. A, AST, CTN-I, and LDH indexes of mice after treatments. **B,** H&E staining of the hearts of the mice with different treatments (black arrows indicate prominent myocardial capillary congestion), scale bar = 50 μm. Masson staining of the hearts of the mice with different treatments (black arrows indicate myocardial fibrosis), scale bar = 50 μm. **C,** H&E staining of the lungs parenchyma and interstitium of the mice with different treatments. Black arrows indicate interalveolar hemorrhage and interstitial fibrosis, scale bar = 50 μm. Data represented as mean ± SD (n = 6). (*p<0.05, **p<0.01, ***p<0.001, ****p<0.0001; NS represents non-significance).

Table S1. Characterization of the CM vesicles, PLGA NPs and PLGA NPs loaded with PTX (n = 3).

Formulation	Size (nm)	PDI	Zeta potential (mV)
CM vesicles	215.2±7.9	0.13±0.01	-25.8±0.1
NP	142.3±3.5	0.12±0.01	-15.0±0.4
PCM@NP	150.7±4.2	0.11±0.01	-24.9±1.3
Anti-CD47-PCM@NP	152.3±3.5	0.12±0.02	-24.4±1.2
NP/PTX	155.5±6.8	0.09±0.01	-13.9±0.3
PCM@NP/PTX	162.3±5.9	0.12±0.02	-19.3±1.2
Anti-CD47-PCM@NP/PTX	168.7±5.5	0.13±0.02	-18.9±1.0

Table S2. Antibody and metal isotopes for CyTOF.

List	Label	Marker	Clone	Manufacturer	Cat No.
1	89Y	CD45	30-F11	BioLegend	103102
2	115In	CD3e	145-2C11	BioLegend	100302
3	141Pr	CD49b	DX5	BioLegend	108902
4	142Nd	CD95(Fas)	SA367H8	BioLegend	152602
5	143Nd	NKp46	29A1.4	BioLegend	137625
6	144Nd	CD40	HM40-3	BioLegend	102902
7	145Nd	CD70	FR70	Thermo	14-0701-82
8	146Nd	CD28	37.51	BioLegend	102102
9	147Sm	Ly6G	1A8	BioLegend	127602
10	148Nd	Ly6C	HK1.4	BioLegend	128002
11	149Sm	CD64	X54-5/7.1	BioLegend	139302
12	150Nd	CD127	A7R34	BioLegend	135002
13	151Eu	CD62L	MEL-14	BioLegend	104402
14	152Sm	CD11c	N418	BioLegend	117302
15	153Eu	TCRgd	GL3	BioLegend	118101
16	154Sm	CX3CR1	SA011F11	BioLegend	149002
17	155Gd	BST2	44E9R	R&D Systems	MAB8660
18	156Gd	CD194(CCR4)	2G12	BioLegend	131202
19	157Gd	CD83	Michel-19	BioLegend	121502
20	158Gd	B220	RA3-6B2	BioLegend	103202
21	159Tb	F4_80	C1:A3-1	Bio-Rad	MCA497G
22	160Gd	TCRb	H57-597	BioLegend	109202
23	161Dy	CD44	IM7	BioLegend	103002
24	162Dy	CD183(CXCR3)	CXCR3-173	BioLegend	126502
25	163Dy	CD25	3C7	BioLegend	101902
26	164Dy	CD103	2E7	BioLegend	121402
27	165Ho	FOXP3	FJK-16s	Thermo	14-5773-82
28	166Er	CD154(CD40L)	MR1	BioLegend	106508
29	167Er	CD206	C068C2	BioLegend	141702
30	168Er	CD27	LG.3A10	BioLegend	124202
31	169Tm	CD69	H1.2F3	BioLegend	104502
32	170Er	T_bet	4B10	BioLegend	644802

List	Label	Marker	Clone	Manufacturer	Cat No.
33	171Yb	CD24	M1/69	BioLegend	101802
34	172Yb	CD80	16-10A1	BioLegend	104702
35	173Yb	CD86	GL-1	BioLegend	105002
36	174Yb	CD192(CCR2)	475301	R&D Systems	MAB55381-100
37	175Lu	CD196(CCR6)	29-2L17	BioLegend	129802
38	176Yb	MHC_II	M5/114.15.2	BioLegend	107602
39	197Au	CD4	RM4-5	BioLegend	100520
40	198Pt	CD8a	53-6.7	BioLegend	100746
41	209Bi	CD11b	M1/70	BioLegend	117302

194Pt：Live/dead

191/193Ir：DNA

Table S3. The expression of specific markers of each cluster.

Cluster	Cell type	Subtype	Key marker	Annotation marker
C01	T cells	CD4T	Ly6C+	TCRb+CD4+
C02	T cells	CD4T		TCRb+CD4+
C03	T cells	CD4T		TCRb+CD4+
C04	T cells	CD4T	CD69-Treg	TCRb+CD4+
C05	T cells	CD4T	CD69+Treg	TCRb+CD4+
C06	T cells	CD4T	CD86+	TCRb+CD4+
C07	T cells	DPT		TCRb+CD4+CD8+
C08	T cells	CD8T		TCRb+CD8+
C09	T cells	CD8T	Ly6C+	TCRb+CD8+
C10	T cells	CD8T	Ly6C+CD69+	TCRb+CD8+
C11	T cells	CD8T	CD69+	TCRb+CD8+
C12	T cells	CD8T	CD40+	TCRb+CD8+
C13	T cells	CD8T		TCRb+CD8+
C14	T cells	DNT	CD69+	TCRb+CD4-CD8-
C15	T cells	gdT	CD103+	TCRgd+
C16	ILC		CD69+	Lin-CD127+
C17	NK		CD69+	NKp46+
C18	NK		CD49b+CD11b+	NKp46+
C19	DC		CD103-	CD11c+MHCII+
C20	DC		CD103+	CD11c+MHCII+
C21	Monocytes		MHCII-	Ly6C+CD11b+
C22	Macrophage		MHCII+	CD11b+CD64+
C23	Macrophage		MHCII+	CD11b+CD64+
C24	Macrophage		MHCII+	CD11b+CD64+
C25	Macrophage		CD24+	CD11b+F4/80+
C26	Macrophage		CD24+	CD11b+F4/80+
C27	MDSC			CD11b+CD44+
C28	MDSC			Ly6G+CD11b+
C29	MDSC			Ly6G+CD11b+
C30	MDSC		CD24-	Ly6G+CD11b+
C31	MDSC			Ly6G+CD11b+
C32	MDSC		Ly6C+	Ly6G+CD11b+

Table S4. Hematotoxicity analysis of Anti-CD47 (Day 15).

	RBC	HGB	HCT	MCV	MCH	MCHC	RDW-CV	RDW-SD	PLT	MPV	PDW	PCT
	10^{12} L^{-1}	g L^{-1}	%	fL	pg	g L^{-1}	%	fL	10^{9} L^{-1}	10^{9} L^{-1}		%
Range	6.00-12.50	100-190	40.0-48.0	41.0-63.0	13.0-19.0	290-351	10.0-20.0	0.1-99.9	540-1540	3.8-14.1	0.1-30.0	0.010-9.990
Saline												
1	6.94	144	45.0	50.5	16.5	328	14.2	28.4	741	6.1	6.2	0.370
2	7.51	144	44.8	46.3	18.2	393	14.3	26.6	814	6.2	6.7	0.520
3	8.28	146	41.6	50.2	18.0	359	13.9	27.7	824	6.8	8.4	0.472
Anti-CD47												
1	5.90	78	27.0	42.6	13.5	300	13.7	252	415	6.4	7.3	0.396
2	4.12	92	32.6	42.5	17.4	332	11.8	25.1	699	6.9	8.9	0.345
3	6.16	88	30.0	43.0	17.0	322	11.5	22.6	467	7.2	8.6	0.336
PCM@NP												
1	8.18	142	41.2	50.3	17.4	346	13.5	27.2	561	6.7	8.4	0.441
2	8.39	151	39.6	47.2	18.0	382	13.0	24.6	560	7.0	7.9	0.393
3	7.67	127	40.3	52.5	16.6	315	18.8	39.8	644	6.5	7.3	0.420
Anti-CD47-PCM@NP												
1	8.49	115	42.1	49.6	16.9	342	13.8	27.2	606	6.4	6.8	0.474
2	8.55	137	42.3	49.5	16.9	341	14.0	27.5	739	6.2	6.7	0.503
3	8.61	149	42.5	49.4	17.0	343	14.0	27.5	691	6.2	6.2	0.514

Table S5. ***In vitro*** **anti-tumor activity test of Anti-CD47-PCM@NP/PTX and other formulations (n = 6).**

Formulation	IC_{50} (ng mL^{-1})
Abr	340.0±10.8
NP/PTX	410.5±35.6
PCM@NP/PTX	160.1±54.1
Anti-CD47-PCM@NP/PTX	118.0±43.7

Table S6. Hematotoxicity analysis of ADC (Day 15).

	RBC	HGB	HCT	MCV	MCH	MCHC	RDW-CV	RDW-SD	PLT	MPV	PDW	PCT
	10^{12} L^{-1}	g L^{-1}	%	fL	pg	g L^{-1}	%	fL	10^{9} L^{-1}	10^{9} L^{-1}		%
Range	6.00-12.50	100-190	40.0-48.0	41.0-63.0	13.0-19.0	290-351	10.0-20.0	0.1-99.9	540-1540	3.8-14.1	0.1-30.0	0.010-9.990
Saline												
1	6.94	115	45.0	50.5	16.5	328	14.2	28.4	606	6.1	6.2	0.370
2	7.51	137	44.8	46.3	18.2	393	14.3	26.6	839	6.2	6.7	0.520
3	8.28	149	41.6	50.2	18.0	359	13.9	27.7	691	6.8	8.4	0.472
ADC												
1	3.68	66	20.6	55.9	17.9	320	11.5	50.4	490	6.5	7.8	0.383
2	2.28	37	10.8	47.5	16.3	342	13.4	27.0	451	6.2	6.2	0.278
3	2.25	59	16.0	49.7	17.2	345	14.0	27.5	458	6.9	8.3	0.457
PCM@NP												
1	8.18	142	41.2	50.3	17.4	346	13.5	27.2	661	6.7	8.4	0.441
2	8.39	151	39.6	47.2	18.0	382	13.0	24.6	560	7.0	7.9	0.393
3	7.67	127	40.3	52.5	16.6	315	18.8	39.8	644	6.5	7.3	0.420
ADC-PCM@NP												
1	9.00	146	41.5	46.1	16.2	351	14.7	26.6	610	8.0	17.3	0.491
2	8.75	152	42.4	48.5	17.4	359	14.4	27.8	898	6.6	7.2	0.590
3	8.89	143	40.6	45.6	16.1	353	14.4	25.8	808	7.3	11.8	0.590

Movie S1. Instrumental field of view for single cell fluorescence signal detection by the Real-time Single cell Multi-modal Analyzer nanoprobe.

Movie S2. Microscopic field of view for single cell fluorescence signal detection by the Real-time Single cell Multi-modal Analyzer nanoprobe.

Movie S3. Free Anti-CD47 in the flowing state to bind to the target cells adsorbed in the cavity of the microfluidic chip.

The target cells (4T1) were adsorbed in the cavity of the microfluidic chip, and then exposed to FITC-labeled free Anti-CD47 in flowing state, and the binding of Anti-CD47 on 4T1 cells was recorded in real-time by a fluorescence microscope. The video shows that Anti-CD47 could bind to the target cells.

Movie S4. *In vitro* transfer of antibody from Anti-CD47-PCM@NP to the surface of 4T1 cells was assessed by microfluidic assay.

The target cells (4T1) were adsorbed in the cavity of the microfluidic chip, and then exposed to Anti-CD47-PCM@NP in a flowing state (antibody was labeled with FITC, and PCM@NP was labeled with DiD), and the fluorescence was recorded in real-time by the fluorescence microscope. The video shows that antibodies were effectively released from Anti-CD47-PCM@NP and accumulated around the target cells membrane, accompanied by substantial internalization of the dissociated PCM@NP.

Movie S5. No significant surface binding of antibody or PCM@NP internalization was observed on CD47$^{-/-}$ 4T1 cells.

The non-target cells (CD47$^{-/-}$ 4T1) were adsorbed in the cavity of the microfluidic chip, and exposed to the Anti-CD47-PCM@NP in a flowing state (antibody was labeled with FITC, and PCM@NP was labeled with DiD), and the fluorescence microscope was recorded in real-time. The video shows that no significant surface binding of antibody or PCM@NP internalization was observed on non-target cells.

Reference

[1] N. Maurer, D. B. Fenske, P. R. Cullis, *Expert opinion on biological therapy* **2001**, 1, 923.
